# DESI-MSI applications for direct (spatial) biomolecular analysis of South African natural medicinal products

**DOI:** 10.3389/fpls.2026.1802351

**Published:** 2026-06-03

**Authors:** Kabelo N. Mohlamonyane, Efficient N. Ncube, Marco Giampà, Weiyang Chen, Dylan Zeiss, Peter D. Verhaert, Bart Ghesquière, Alvaro M. Viljoen

**Affiliations:** 1 Department of Pharmaceutical Sciences, Faculty of Science, Tshwane University of Technology, Pretoria, South Africa; 2 South Africa Medical Research Council (SAMRC) Herbal Drugs Research Unit, Tshwane University of Technology, Pretoria, South Africa; 3 Laboratory for Applied Mass Spectrometry (LAMaS), Department Cellular and Molecular Medicine, University of Leuven, Leuven, Belgium; 4 Waters Division, Microsep (Pty) Ltd., Sandton, South Africa; 5 ProteoFormiX, Vorselaar, Belgium; 6 Integrated Molecular Physiology Research Initiative, University of the Witwatersrand, Johannesburg, South Africa; 7 Metabolomics Core Leuven, Vlaams Instituut voor Biotechnologie (VIB) Technologies, VIB, Leuven, Belgium

**Keywords:** DESI-MSI, *in situ* analysis, medicinal plants, metabolite profiling, natural products, pharmacognosy, quality control, spatial distribution

## Abstract

**Introduction:**

In pharmacognosy, conventional analytical methods provide important chemical information on metabolite identities and relative quantities. However, *in situ* analysis and the mapping of the spatial distribution of metabolites remain underexplored. Mass spectrometry (MS) imaging techniques, specifically desorption electrospray ionization-MS imaging (DESI-MSI), enables the *in situ* analysis and visualization of the spatial distribution of metabolites in different plant tissues. This study aimed to demonstrate the application of DESI-MSI for the *in situ* analysis and visualization of the spatial distribution of metabolites in selected South African medicinal plants and natural products.

**Methods:**

DESI-MSI was used to putatively assign antimicrobial compounds from propolis *via* HPTLC-bioautography assay and to visualize the spatial distribution of various compounds, including coumarins in the roots of *Pelargonium reniforme* and *Pelargonium sidoides*, pyrrolizidine alkaloids in the stems of *Lobostemon fruticosus*, and aspalathin in the leaves of *Aspalathus linearis.*

**Results:**

The antimicrobial compounds of South African propolis were putatively assigned as chrysin, pinocembrin, galangin, pinobanksin and its derivatives. The technique enabled the differentiation between *P. sidoides* and *P. reniforme* based on the location and spatial distribution of umckalin, dihydroxy-dimethoxycoumarin and isofraxidin sulphite in the root samples. Furthermore, the spatial distribution of pyrrolizidine alkaloids (PAs) was mapped in the pith, epidermis, and cortex regions of *L. fruticosus* stems, while aspalathin was mapped in the margins and tips (upper and lower epidermal regions) of *A. linearis* leaf samples.

**Conclusion:**

This study demonstrates the feasibility of using DESI-MSI for *in situ* chemical profiling of commercially important South African medicinal plants and natural products, supporting their identification and differentiation for quality control.

## 1 Introduction

According to the World Health Organization (WHO), approximately 21,000 plants possess the capacity of being used as medicinal plants (World Health Organization, 2019; Heinrich and Anagnostou, 2017). In South Africa, it has been reported that over 27 million people rely on traditional medicine for their health-related needs due to accessibility, comprehensive knowledge, and cost effectiveness (Efferth and Greten, 2012; Street et al., 2008). Many of these medicinal plants in South Africa are naturally sourced and tend to display chemotypic variation in their chemical composition which could potentially reduce their therapeutic efficacy (Street et al., 2008). Conventional analytical techniques like gas chromatography (GC), high performance liquid chromatography (HPLC), ultra-performance liquid chromatography (UPLC) hyphenated to optical and mass spectrometry (MS) detection, mid-infrared (MIR), near-infrared (NIR), nuclear magnetic resonance spectroscopy (NMR), and fluorescence transmission-infrared spectroscopy (FT-IR) (Abubakar and Haque, 2020; Jiang et al., 2021) can characterize metabolites, but lack the capabilities to map the spatial distribution within plant tissues (Cao et al., 2024; Chen et al., 2024).

The South African medicinal plants investigated in this study were selected based on their clinical importance and unresolved research questions. Propolis, a resin-based product, has demonstrated efficacy against respiratory tract infections due to its high flavonoid content (galangin, chrysin, and pinocembrin) (Cushnie and Lamb, 2005; Kharsany, 2019; Rahman et al., 2010; Suleman, 2015). However, the bioactive compounds of SA propolis against *Streptococcus pneumoniae* remain poorly characterized. The roots of *Pelargonium reniforme* and *Pelargonium sidoides* are used interchangeably to treat respiratory and gastrointestinal ailments due to their morphological similarity. However, the roots of these species display coumarin variation (Kolodziej, 2007; Maree and Viljoen, 2011, MareeViljoen, 2012), and the spatial distribution of these chemomarkers have not been mapped. *Lobostemon fruticosus* is traditionally used for wound healing but contain potentially hepatotoxic PAs compounds. The *in situ* localization of these toxic compounds remains unknown. Lastly, *Aspalathus linearis* (rooibos tea) produces aspalathin, a C-glucosyl dihydrochalcone linked to the antioxidant properties of the commercial tea (Amor Stander et al., 2019). The abundance and distribution of aspalathin between young and mature tissues varies. While this compositional difference is well documented, the *in situ* spatial distribution of this compound in different developmental stages has not been reported (Bednarska and Fecka, 2022; Joubert and de Beer, 2011).

DESI-MSI offers a simple and distinctive approach in pharmacognosy-based studies (Müller et al., 2011). This technique allows the ionization of different chemical compounds under ambient environment, and suitable for the minimally destructive and rapid analysis of a diverse range of samples including plant material and natural products (Abbassi-Ghadi et al., 2015; Huang et al., 2011; Rankin-Turner et al., 2023). Additionally, compatibility with bioactivity assays enables direct correlation of spatial metabolite data with therapeutic effects (Parrot et al., 2018). DESI-MSI provides a complementary analytical approach to established techniques such as MALDI-MSI and LC-MS-based metabolomics. While LC-MS provides effective compound separation and identification, DESI-MSI can advance natural products research by enabling direct, spatially resolved chemical analysis of different plant matrices with minimal sample preparation. In addition, MALDI-MSI typically requires matrix application (Abbassi-Ghadi et al., 2015; Huang et al., 2011; Rankin-Turner et al., 2023; Tillner et al., 2017).

In this proof-of-concept study, DESI-MSI is applied as a versatile and innovative analytical approach linking spatial metabolite distribution with diverse pharmacognosy-based applications of South African natural medicinal products. The application of DESI imaging in natural products research was demonstrated by (i) the rapid identification of antimicrobial flavonoids in propolis directly from inhibition zones on bioautographic HPTLC plates, (ii) mapping the distribution of metabolites in the roots of *Pelargonium* species for chemotaxonomic classification, (iii) assessing the presence and localization of pyrrolizidine alkaloids in the stems of *L. fruticosus* for safety assessment and (iv) compare the spatial distribution of aspalathin in young versus mature green rooibos leaves to evaluate the effects of product processing. Together, these case studies highlight DESI imaging as a tool for rapid and effective functional metabolite profiling, chemotaxonomy, and quality control for natural products-based research.

## 2 Materials and methods

### 2.1 Chemicals and reagents

Ethanol and formic acid were obtained from Thembane Chemicals (Johannesburg, South Africa). Ethyl acetate and toluene were purchased from Merck Millipore (Johannesburg, South Africa). Haemophilus Test Medium (HTM), HTM Supplement SR0158, *Streptococcus pneumoniae* (ATCC 6841) was sourced from University of Witwatersrand (Johannesburg, South Africa), super pure-grade methanol was obtained from Romil (Johannesburg, South Africa).

### 2.2 Sample collection

The propolis, collected from Muldersvlei (Stellenbosch, Western Cape province, South Africa), was available from a previous study (van Vuuren, University of the Witwatersrand). The roots of *P*. *reniforme* and *P. sidoides*, stem of *L. fruticosus*, and leaves (young and mature) from *A. linearis* were sourced from Parceval (Pty) Ltd., South Africa. The samples were stored in the Department of Pharmaceutical Sciences, Tshwane University of Technology.

### 2.3 Sample preparation

#### 2.3.1 Propolis extraction

A mass of 50 mg crude extract of propolis was immersed in 1 mL of ethanol and subjected to an orbital shaker (Labcon, South Africa) for 24 h at room temperature. The sample was sonicated in an ultrasonic bath (LIBM8, Labcon^®^, South Africa) at 30 °C for 10 minutes. The final suspension was filtered through a 0.22 μm syringe filter (Agela technologies, USA). The sample was stored at 4 °C until ready for analysis.

#### 2.3.2 *Pelargonium* roots and *L. fruticosus* stem sectioning

The roots of *P. reniforme* and *P. sidoides*, and stem of *L. fruticosus* were washed and stored in -80 °C freezer until use. The frozen tissues were stabilized for 1 h at -20 °C in a Dakewe 6250 cryostat microtome (Cell path services, South Africa). The root tissues were embedded on a sample holder (Specimen chuck) using deionized water. The specimen chucks were placed on the freezing stage, and the tissues were sectioned (80 µm) slice at -20 °C as outlined by Gao and Li (2023) and Xia et al. (2023). The tissues were mounted on a glass slide (Lasec, South Africa) for DESI-MSI analysis.

#### 2.3.3 *Aspalathus linearis* leaf imprinting

The leaves from *A. linearis* (young vs mature) were prepared using the so called paper imprinting method: the leaf tissues were placed on the top of a filter paper (Lasec, South Africa) and covered with another layer of tissue paper and placed between two aluminum plates, covered with rubber sheets, and compressed with bench-vice clamp for 30 seconds (Wu et al., 2022). The leaf-imprinted paper was affixed to a glass slide using double-sided tape for direct analysis by DESI-MSI.

#### 2.3.4 *Streptococcus* culture

The medium was prepared by suspending 21.5 g of Haemophilus Test Medium (HTM) (Thermo Fisher Scientific, South Africa) in 500 mL of sterile distilled water. Thereafter, the mixture was autoclaved at 121 °C for 15 minutes. The media was cooled to 50 °C, and one vial of HTM Supplement SR0158 was aseptically added and mixed well. *Streptococcus pneumoniae* (ATCC 6841) was cultured in the media and incubated in a CO_2_ incubator for approximately 18–24 hours at 37 °C. After incubation, the bacterial cultures were standardized by diluting 1:100 using HTM agar to achieve the 0.5 McFarland turbidity standard.

### 2.4 HPTLC analysis

The analysis was conducted using a semi-automated HPTLC system supplied by CAMAG (Muttenz, Switzerland) consisting of an automatic sampler 4, chromatogram immersion device, automatic developing chamber (ADC 2), TLC plate heater and a documentation device (*vision*CATS) used for operating parameters and data analysis. A volume of 2 µL of 50 mg/mL of propolis extract was applied on pre-coated silica gel 60 F_254_ glass plates (20 × 10 cm) as 10 mm band, applied 10 mm from the bottom of the edge and 10 mm from the edge side, with a delivery speed of 150 nL/s using an auto sampler fitted with 25 µL Hamilton syringe. The plate was air-dried for 3 minutes prior to development. The developing chamber was conditioned to 33% relative humidity using a saturated aqueous magnesium chloride solution for 10 minutes. The plates were developed to a migration distance of 80 mm in a pre-saturation chamber for 20 minutes using toluene: ethyl acetate: formic acid (78:22:5 v/v/v) (Sankaran et al., 2023). The plates were air-dried for 5 minutes and visualized under white reflectance and UV 366 nm radiation prior to derivatization. The *p*-Anisaldehyde reagent was prepared by adding 85 mL of methanol, 10 mL of acetic acid, 5 mL of sulphuric acid, and 0.5 mL of anisaldehyde. The reference plate was sprayed with 3 mL of *p*-Anisaldehyde reagent in a TLC spraying chamber. After spraying, the HPTLC plate was visualized under white reflectance and UV 366 nm radiation. The plate images were analyzed using winCATS^®^ software (Mulaudzi, 2020).

### 2.5 Bioautography assay

The HPTLC plates were sterilized for 30 minutes under blue visible light. A volume of 100 mL of agar medium was poured into square petri dish with 25 x 25 cm dimension and allowed to solidify. The sterilized HPTLC plates were placed in the center of the petri dish facing up and were covered with a volume of 100 mL of agar inoculum at approximately 40 °C to attain the bioautogram. The agar was allowed to solidify, and the plates were stored at –4 °C for 30 minutes to stabilize the medium. Thereafter, the plates were incubated for 18–24 hours at 37 °C in a CO_2_ incubator. After incubation, the bio-autographs were sprayed with 0.4 mg/mL of *p*-iodonitrotetrazolium violet solution (INT) at room temperature to visualize inhibition zones. The plates were further incubated for 4–6 hours at room temperature until a red color developed to indicate microbial growth. The Rf values of the zones were determined and correlated with the Rf values of the bands observed on the reference HPTLC plate under UV/visible light.

### 2.6 DESI-MSI analysis

The imaging analysis was performed on a Xevo™ G3 QToF mass spectrometer (Waters Corporation, Manchester, UK) equipped with a DESI-XS source, containing heated transfer line (HTL) or an ion inlet tube, high-performance DESI sprayer and a 2D moving stage for imaging. Prior to data acquisition, instrumental conditions were refined through iterative adjustment of sprayer geometry, solvent composition, flow rate, and ion source parameters to achieve a stable signal and optimal analytical performance, in accordance with manufacturer-recommended guidelines for the DESI XS platform. Such parameter considerations are known to influence ionization efficiency, spatial resolution, and signal stability in DESI-MSI analyses (Tillner et al., 2017). The selected conditions were further informed by established DESI-MSI applications in plant and biological systems, with final values adapted to suit the specific sample matrices analyzed (Müller et al., 2011; Kumara et al., 2019; Xia et al., 2023). The DESI-MSI parameters utilized for all samples were as follows: capillary voltage, 0.70–0.80 kV; ion source temperature, 120 °C; nitrogen pressure, 0.1 MPa; capillary tip to the surface, 2 mm; capillary tip to MS inlet, 6 mm; MS inlet to surface, ~ 0.5 mm; spray solvent, methanol: water (98:2) with 0.01% formic acid, containing leucine enkephalin of 50 pg/µL (internal standard for mass correction). The flow rate was set at 3 µL/min. Harvard Apparatus system (Holliston, USA) was used to deliver the solvent with a 1.00 mL syringe (Microsep, South Africa). Other sample-specific parameters are mentioned in **Table 1**. Spectrum data were acquired using sample-specific, pixel sizes, and scan rates. A mass range of *m/z* 100–1200 was used. Sample-specific ionization modes were selected based on the chemical classes of interest, with negative ionization mode applied for *Pelargonium* spp. and propolis samples to enhance detection of phenolic compounds, and positive ionization mode used for *L. fruticosus* and *A. linearis* to facilitate detection of alkaloids and flavonoid derivatives. Routine maintenance of the DESI source components, including cleaning of the ion inlet, sprayer nozzle, heated transfer line, and emitter cartridge, was performed to ensure consistent analytical performance (Tillner et al., 2017).

**Table 1:** DESI-MSI acquisition parameters used for the analysis of each sample.

Parameters	Propolis	*Pelargonium spp*	*L. fruticosus*	*A. linearis*
**Ionisation mode**	Negative ion mode	Negative ion mode	Positive ion mode	Positive ion mode
**Spray angle**	74°	76°	60°	66°
**Sample cone voltage**	15 V	30 V	30 V	40 V
**MS inlet configuration**	Ion inlet tube	120 °C HTL	Ion inlet tube	120 °C HTL
**Imaging acquisition (x, y)**	X= 400 Y= 400	X= 250 Y= 250	X= 200 Y= 200	X= 200 Y= 200
**Acquisition Rate**	Rate = 1500 µm/s	Rate = 400 µm/s	Rate = 800 µm/s	Rate = 800 µm/s
**Acquisition time (imaging duration)**	50 min	125 min	23 min	31 min

Peak annotation (putative compound identification) was performed based on high-resolution accurate mass measurements and comparison with literature data. To support compound identification, complementary UPLC-QToF-MS analysis was performed for all samples, including some reference standards where available, under optimized chromatographic and mass spectrometry conditions provided level 1 and 2 (≤10 ppm) (full details in the **Supplementary Material**). To confirm the peak assignments, MS spectra for representative compounds, include proposed elemental compositions, double bond equivalents (DBE), calculated masses, and mass accuracy (ppm).

### 2.7 Data processing

High-definition imaging (HDI) 1.8 (Waters Corporation, Manchester, UK) software was utilized for the processing and visualization of the raw image data files. During pre-processing, the acquired data was lock-mass corrected to the reference mass of leucine enkephalin using a mass accuracy tolerance of 10 ppm and signal intensity tolerance of 1000 counts per second. To account for variability in signal intensity across pixels and differences in sample heterogeneity, all DESI-MS ion images were normalized to the total ion current (TIC), where each pixel’s ion intensity was expressed as a fraction of the total ion signal detected at that location. This normalization is widely applied in mass spectrometry imaging studies (Hemalatha and Pradeep, 2013; Tillner et al., 2017). The acquired *m/z* values of the metabolites were converted into 2D ion images using a color intensity scale. The relative ion intensity represented by the variation in color intensity reflected the spatial distribution visualization of the detected compounds (Hemalatha and Pradeep, 2013). The blue color indicated low relative concentration/intensity, while the yellow indicated relative high concentration/intensity for the spatial distribution of the *m/z* in the tissues, with intensity levels in the order of yellow > red > blue (Wang et al., 2024).

For the multivariate analysis to differentiate *Pelargonium* spp, the raw data were converted to HDF5 format and imported into Mozaic (version 2025.12.0.b3). The dataset was subsequently recalibrated using leucine enkephalin as a lock mass at *m/z* 554.2619 [M–H] ^-^. The recalibrated dataset was then converted to imzML, normalized using total ion current (TIC), and peak-aligned based on a reference peak list, containing the *m/z* values of identified compounds, with a tolerance of 50 ppm using the Cardinal R package. Pixel-wise principal component analysis (PCA) was performed, and score and loading plots were generated (Bemis et al., 2015). For the box-and-whisker plots comparing signal intensities of aspalathin in young and mature rooibos leaves, ROI-based intensity values were extracted as multivariate analysis (MVA) data and exported as Excel files. The data were subsequently processed in Jupyter notebook (Version 7.3.2) to run Python using the Pandas library for data handling and transformation. The dataset was converted into long format to facilitate statistical comparison between groups. The box-and-whisker plots were generated, and data visualization was performed using Seaborn and Matplotlib, illustrating the median, interquartile range, and overall variability.

## 3 Results and discussion

### 3.1 HPTLC- DESI-MSI identifies antimicrobial flavonoids in propolis

**Figure 1:**
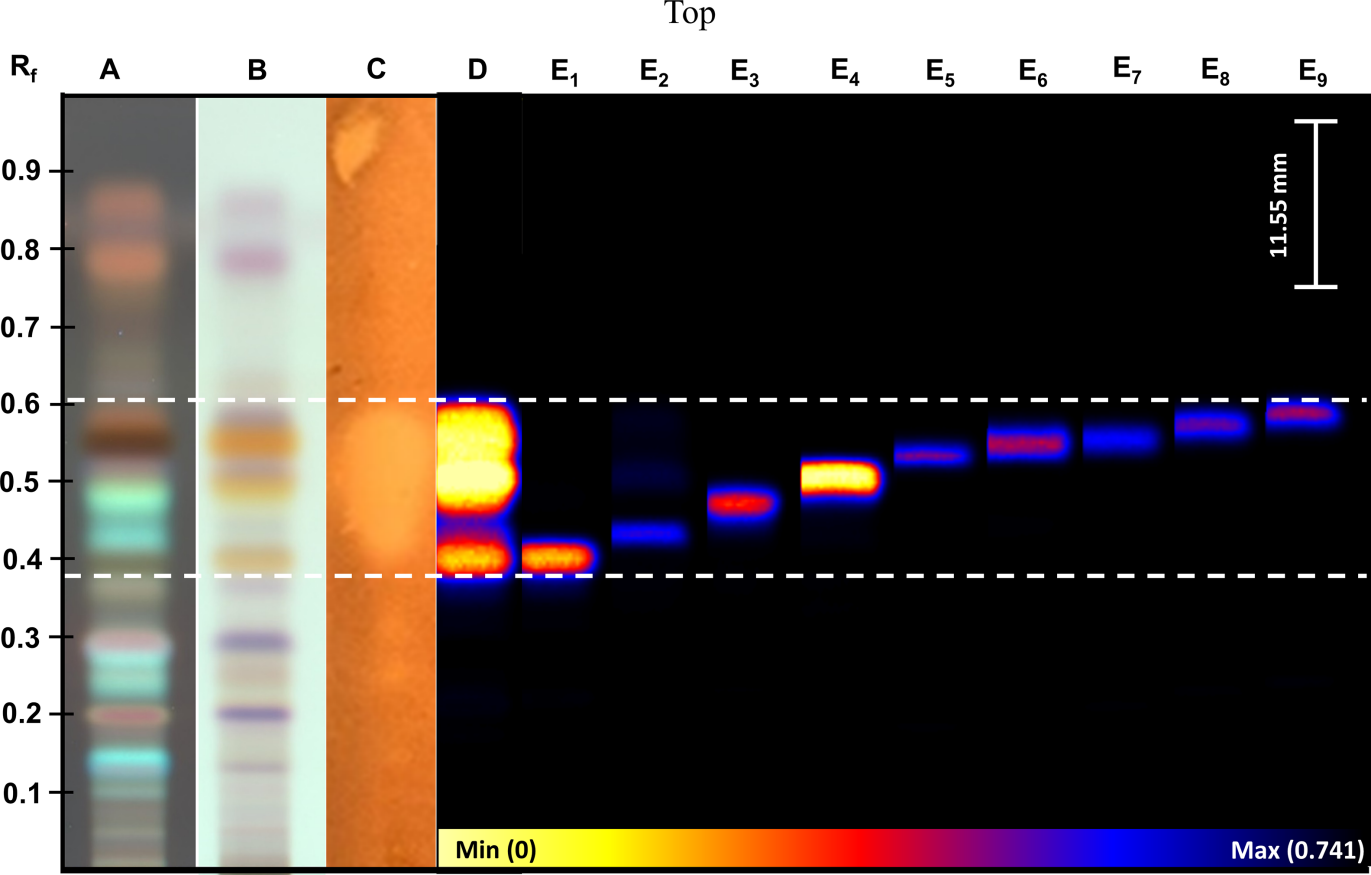
High-performance thin-layer chromatography (HPTLC) analysis of South African propolis extract. The representative plate was visualized under **(A)** UV 366 nm, **(B)** white light reflectance, and **(C)** a bioautogram showing the antibacterial activity of selected bands against *S. pneumoniae*. **(D)** a DESI-MSI overlay of all bands present within the antibacterial region. **(E)**_1_–**(E)**_9_ DESI–MS images of selected chromatographic bands, identified as: **(E)**_1_, Pinobanksin; **(E)**_2_, Chrysin; **(E)**_3_, Galangin; **(E)**_4_, Pinobanksin acetate; **(E)**_5_, Pinobanksin propionate; **(E)**_6_, Pinocembrin; **(E)**_7_, Pinobanksin butyrate; **(E)**_8_, Pinobanksin pentanoate; and E_9_, Pinobanksin hexanoate. Ion intensity signals were normalized to the total ion current and displayed as a heat map. Scale bar = 11.55 mm.

The antimicrobial activity of South African propolis against *S. pneumoniae* was demonstrated through HPTLC-bioautography (**Figures 1A–E**), where a white inhibition zone was observed between R_f_ values between 0.38 and 0.60 (**Figure 1C**). The summary of antimicrobial compounds, along with their associated mass spectral information and corresponding R_f_ values, were listed in **Table 2**.

**Table 2:** Putative identification of antimicrobial compounds in propolis against *S. pneumoniae*.

#	R_f_ value	HPTLC bands colours (UV 366 nm)	HPTLC bands colours (white light)	Calculated mass [M-H]^-^ m/z	Elemental Composition	Compound Name
**E_1_**	0.40	Brown	Orange	271.0620	C_15_H_12_O_5_	Pinobanksin
**E_2_**	0.43	Blue	Orange	253.0511	C_15_H_10_O_4_	Chrysin
**E_3_**	0.48	Blue to yellow	Orange to brown	269.0392	C_15_H_10_O_5_	Galangin
**E_4_**	0.50	Blue	Purple	313.0645	C_17_H_14_O_6_	Pinobanksin acetate
**E_5_**	0.54	Blue	Brownish yellow	327.0798	C_18_H_16_O_6_	Pinobanksin propionate
**E_6_**	0.55	Dark brown	Orange	255.0659	C_15_H_12_O_4_	Pinocembrin
**E_7_**	0.56	Dark brown	Orange	341.1025	C_19_H_18_O_6_	Pinobanksin butyrate
**E_8_**	0.58	Brown	Purple	355.1180	C_20_H_20_O_9_	Pinobanksin pentanoate
**E_9_**	0.60	Brown	Purple	369.1342	C_21_H_22_O_6_	Pinobanksin hexanoate

The putative identity of the propolis derived compounds were confirmed by comparing the MS imaging data with the scientific literature (Falcão et al., 2013; Kasote et al., 2014; Pellati et al., 2011; Widelski et al., 2023). The imaging analysis of the HPTLC plate putatively assigned nine bioactive flavonoids (**Figure 1E**, **Table 2**), including chrysin (*m/z* 253.0511, R*_f_* 0.43), pinocembrin (*m/z* 255.0659, R*_f_* 0.55) and galangin (*m/z* 269.0392, R*_f_* 0.48) (**Figure E_1_-E_3_**). Notably, a group of pinobanksin derivatives (R*_f_* 0.50-0.60) including pinobanksin acetate (*m/z* 313.0645), pinobanksin propionate (*m/z* 327.0798), pinobanksin butyrate (*m/z* 341.1025), pinobanksin pentanoate (*m/z* 355.1180) and pinobanksin hexanoate (*m/z* 369.1362) were identified (**Figure 1E_4_-E_9_**). While these compounds have earlier been detected in propolis via conventional methods (Falcão et al., 2013; Kasote et al., 2014), their specific activity against *S. pneumoniae* represents a novel finding. The antimicrobial mechanisms of these flavonoids in propolis have been previously outlined in literature (Cushnie and Lamb, 2011; De Rossi et al., 2025; Pepeljnjak and Kosalec, 2004). Galangin is known to disrupt bacterial membrane integrity (Echeverría et al., 2017), while pinocembrin induces metabolic stress leading to cell lysis (Rasul et al., 2013). The identified pinobanksin derivatives potentially inhibit ATP synthesis as related compounds (Przybyłek and Karpiński, 2019).

These compounds have previously been reported against *S. aureus* and other microbes (Melliou and Chinou, 2004; Kasote et al., 2015; Ristivojević et al., 2016), however, this study represents the first to document their efficacy against *S. pneumoniae*, expanding the known antimicrobial spectrum of propolis flavonoids. Overall, our findings highlight that DESI-MSI can be an effective method for rapid identification of bioactive compounds from crude extracts of natural products. Also, using this approach, it is also evident that marker compounds from the HPTLC plate can be analyzed directly from the HPTLC plate without standard compounds. It should be noted that analyte diffusion during the agar overlay step in HPTLC-bioautography may influence the spatial fidelity of inhibition zones. However, the approach remains suitable for correlating bioactive regions with associated chemical constituents.

To complement the qualitative HPTLC-bioautography findings, the antimicrobial activity of the propolis extract was quantitatively evaluated using a broth microdilution assay (details in supplementary folder). The extract exhibited an average minimum inhibitory concentration (MIC) of 391 µg/mL against *Streptococcus pneumoniae*.

### 3.2 DESI spatial metabolomics of root cross sections discriminates *Pelargonium* species

**Figure 2:**
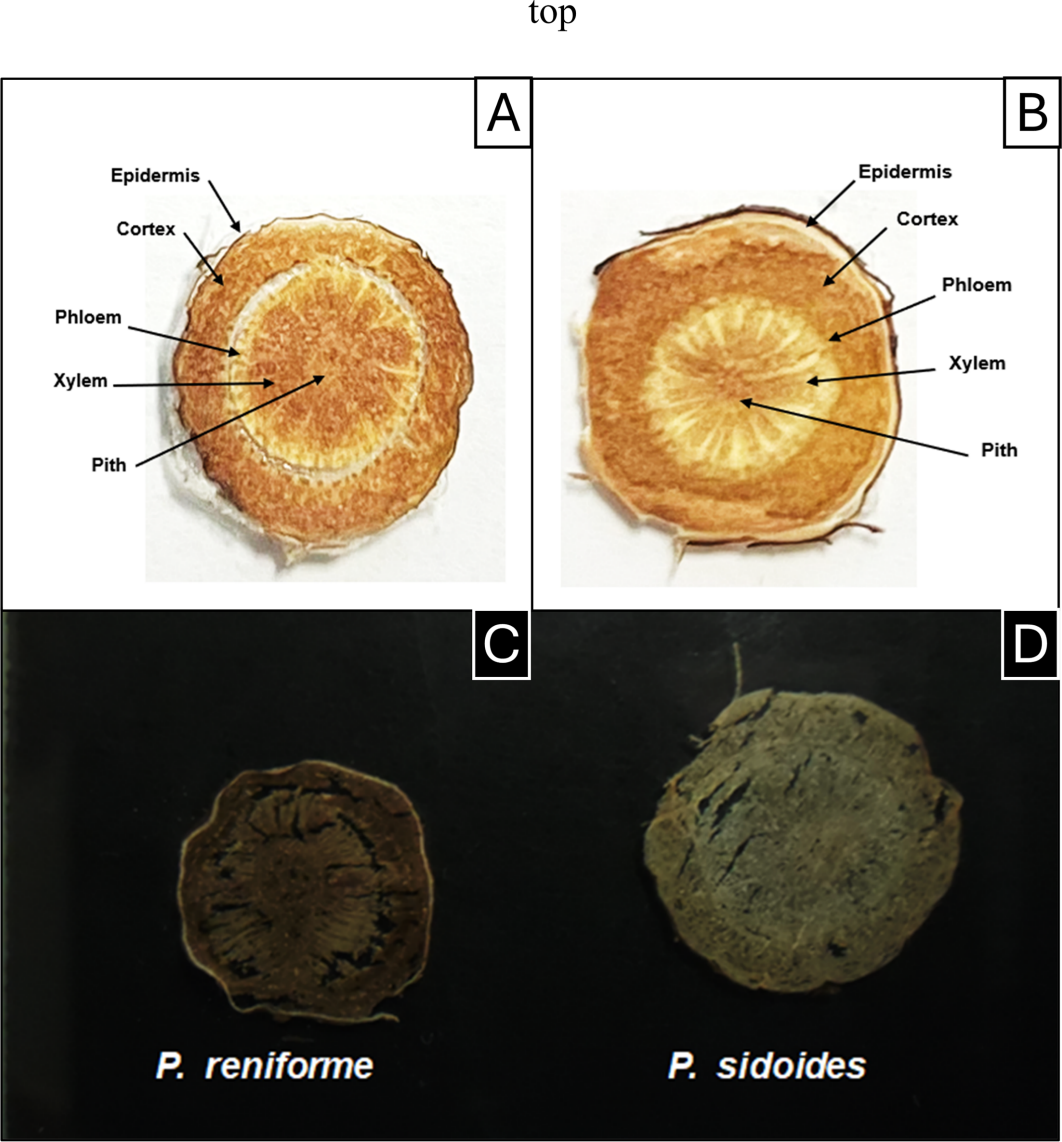
Transverse sections of root tissues from *P. reniforme* **(A, C)** and *P. sidoides* **(B, D)**, highlighting the anatomical regions (epidermis, cortex, phloem, xylem, and pith) found in root tissue.

The distinct and well-defined histology of *Pelargonium* root tissues, comprising clearly defined epidermal, cortical, and vascular regions (**Figures 2A–D**), provided the morphological framework for biomolecular spatial metabolite analysis.

**Figure 3:**
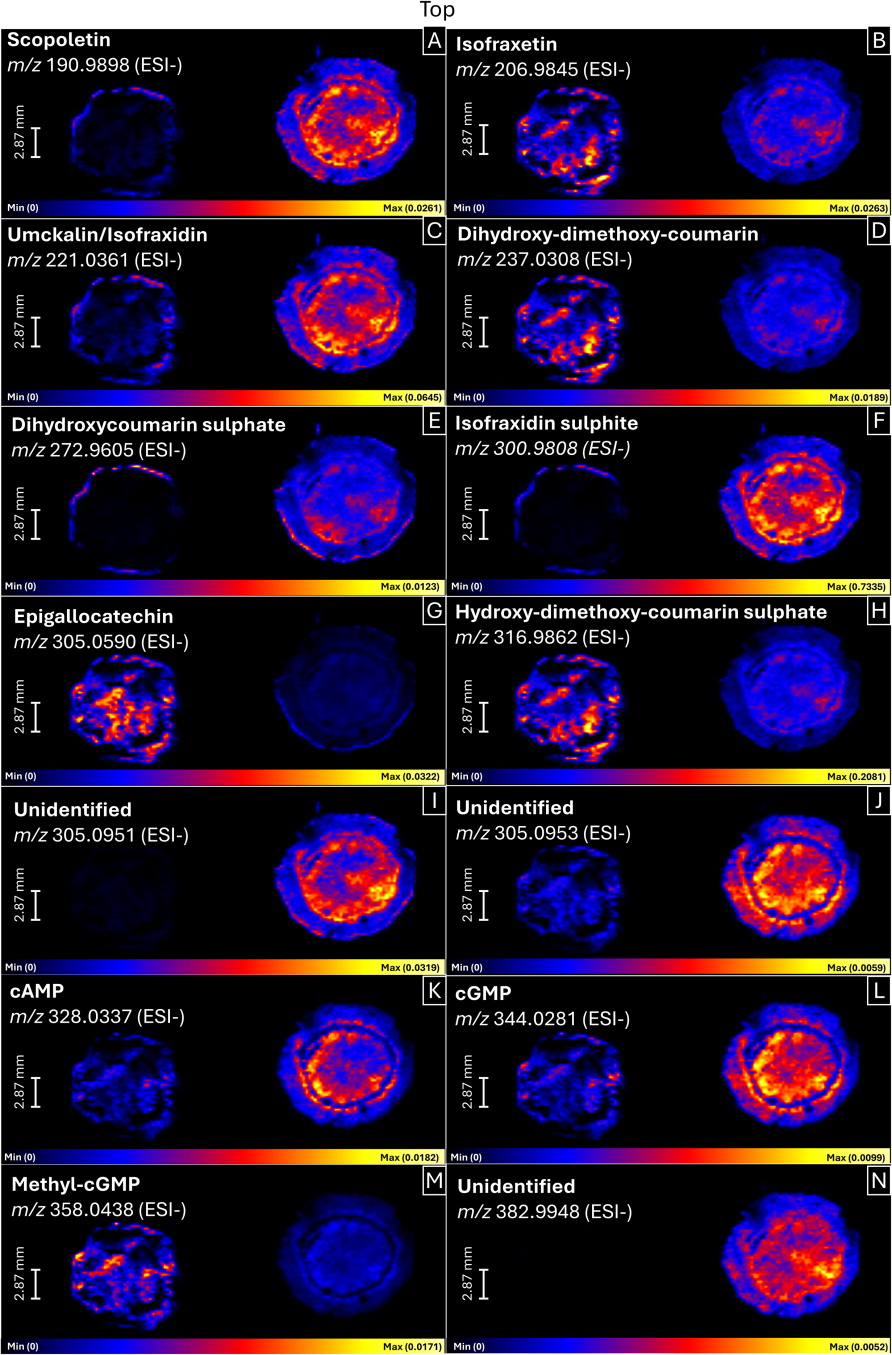
Comparative DESI-MSI images of transverse root tissues from *P. reniforme* (left) and *P. sidoides*, (right) showing the spatial distribution of key metabolites across anatomical regions (epidermis, cortex, xylem, phloem, and pith). The DESI-MSI images are shown for **(A)** scopoletin, **(B)** isofraxetin, **(C)** umckalin/isofraxidin, **(D)** dihydroxy-dimethoxy coumarin, **(E)** Dihydroxycoumarin sulphate, **(F)** isofraxidin sulphite, **(G)** epigallocatechin, **(H)** hydroxy-dimethoxycoumarin sulphate, **(K)** cAMP, **(L)** cGMP, **(M)** methyl-cGMP. Compounds **(I, J, N)** remained unidentified. Ion intensity signals were normalized to the total ion current and displayed as a heat map. Scale bar = 2.87 mm.

The putative identities of the compounds were putatively annotated by comparing accurate *m/z* values with literature data (Kolodziej, 2007; Panara et al., 2022; Qasaymeh, 2021), as shown in **Figure 3**, **Table 3**. DESI-MSI analysis revealed clear differences in metabolite distributions including coumarins between *P. sidoides* and *P. reniforme*. The coumarins isofraxetin (*m/z* 206.9845), umckalin/isofraxidin (*m/z* 221.0361), and dihydroxy-dimethoxycoumarin (*m/z* 237.0399) were revealed as key chemomarkers for species differentiation as reported in previous reports (Kolodziej, 2007; Viljoen et al., 2015). In addition, the detection of modified coumarin derivatives, including sulphated forms, is consistent with the previously described phytochemical diversity of *Pelargonium* species, where coumarins and their derivatives serve as important chemotaxonomic markers (Maree and Viljoen, 2011; Viljoen et al., 2015). The annotation of the respective ions was supported by high-resolution accurate mass measurements and agreement with literature-reported profiles (supplementary folder) (Panara et al., 2022; Qasaymeh, 2021). Importantly, these findings reveal the precise tissue-specific accumulation patterns of these metabolites with previously unattainable spatial resolution.

**Table 3:** Putatively identified compounds in *Pelargonium* species and their spatial distribution in root transverse sections.

Compound Key	Calculated mass [M-H]^-^ *m/z*	Elemental composition	Compound name	Distribution in *P. reniforme*	Distribution in *P. sidoides*
**A**	190.9898	C_10_H_8_O_4_	Scopoletin	Ep	All regions
**B**	207.9845	C_10_H_8_O_5_	Isofraxetin	Xy, Ph, Co, and Ep	Pi, Xy, and Ph
**C**	221.0361	C_11_H_10_O_5_	Umckalin/Isofraxidin	Ep	All regions
**D**	237.0308	C_11_H_10_O_6_	Dihydroxy-dimethoxycoumarin	Xy, Ph, Co, and Ep	Pi, Xy, and Ph
**E**	272.9605	C_9_H_6_O_8_S	Dihydroxycoumarin-sulphate	Ep	Pi, Xy, Ph, and Ep
**F**	300.9808	C_11_H_10_O_8_S	Isofraxidin sulphite	Ep	Pi, Xy, Ph, and Ep
**G**	305.0590	C_15_H_14_O_7_	Epigallocatechin	Pi, Xy, and Ph	Pi, Xy, and Ph
**H**	316.9862	C_11_H_10_O_9_S	Hydroxy-dimethoxycoumarin-sulphate	Ep, Xy, and Ph	Low intensity in all regions
**I**	305.0951	**-**	Unidentified	Low intensity in all regions	Pi, Xy, Ph, and Ep
**J**	305.0953	**-**	Unidentified	Low intensity in all regions	High intensity in all regions
**K**	328.0337	C_10_H_12_N_5_O_6_P	cAMP	Low intensity in all regions	Pi, Xy, and Ph
**L**	344.0281	C_10_H_12_N_5_O_7_P	cGMP	Low intensity in all regions	High intensity in all regions
**M**	358.0438	C_11_H_14_N_5_O_7_P	Methyl-cGMP	Co, Ph, and Xy	Low intensity in all regions
**N**	382.9948	**-**	Unidentified	No detection	High intensity in all regions

*Ep, Epidermis; Xy, Xylem; Ph, Phloem; Co, Cortex; Pi, pith.

**Figure 4:**
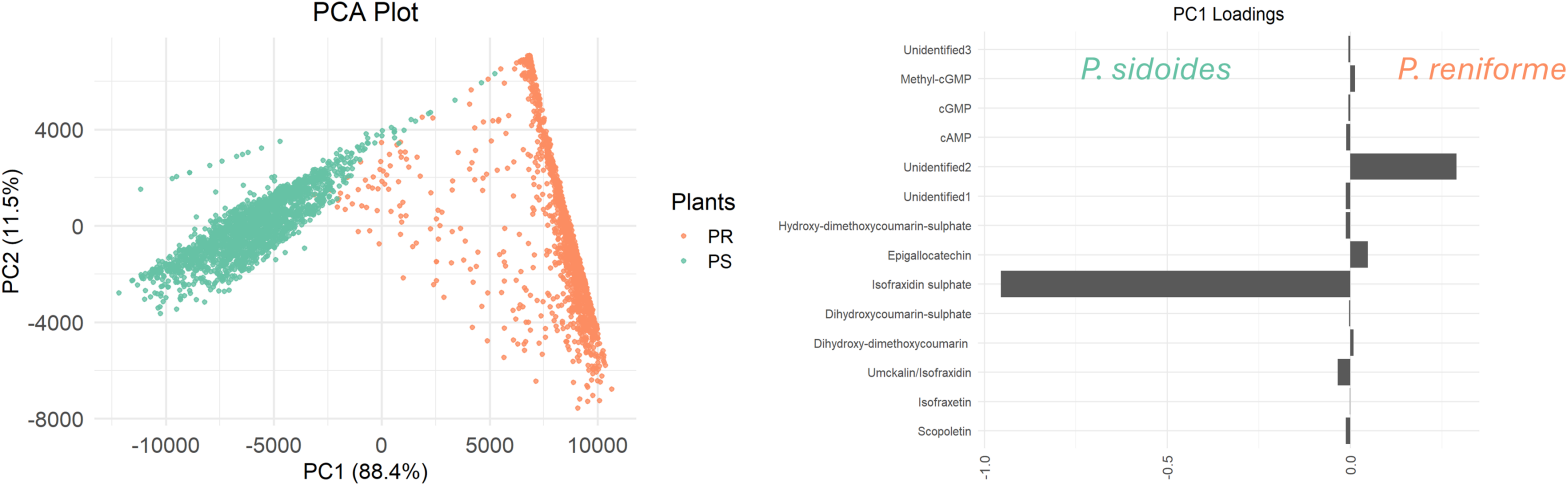
Comparison between *P. sidoides* (PS) and *P. reniforme* (PR). (Left). Single pixel-wise PCA showing two main clusters separation along the PC1 (*P. sidoides* and *P. reniforme* yield negative and positive scores, respectively). (Right). Loadings plots of PC1 showing differentiation between *Pelargonium* species based on different level of targeted metabolites (see e.g., isofraxidin sulphite and epigallocatechin, more correlated to, respectively, *P. sidoides* and *P. reniforme*.

Umckalin and other coumarin derivatives, known for their antimicrobial properties (Kayser and Kolodziej, 1997; Mativandlela et al., 2006), predominated the inner tissues (pith, xylem, and phloem) of *P. sidoides* roots. In contrast, coumarins known for their role as first-line defence compounds against pathogens and herbivores (Mativandlela et al., 2006) were found in relative abundance in the *P. reniforme* roots. Several newly detected coumarin sulphates, including dihydroxycoumarin sulphate (*m/z* 272.9605) and hydroxy-dimethoxycoumarin-sulphate (*m/z* 316.9682), were distributed across the different species as shown in **Table 3**. The chemical differentiation between *P. reniforme* and *P. sidoides*, is further validated by pixel wise PCA of the combined dataset. Score and loading plots are shown in **Figure 4**.

### 3.3 Tissue-specific toxin mapping in *L. fruticosus* stem

**Figure 5:**
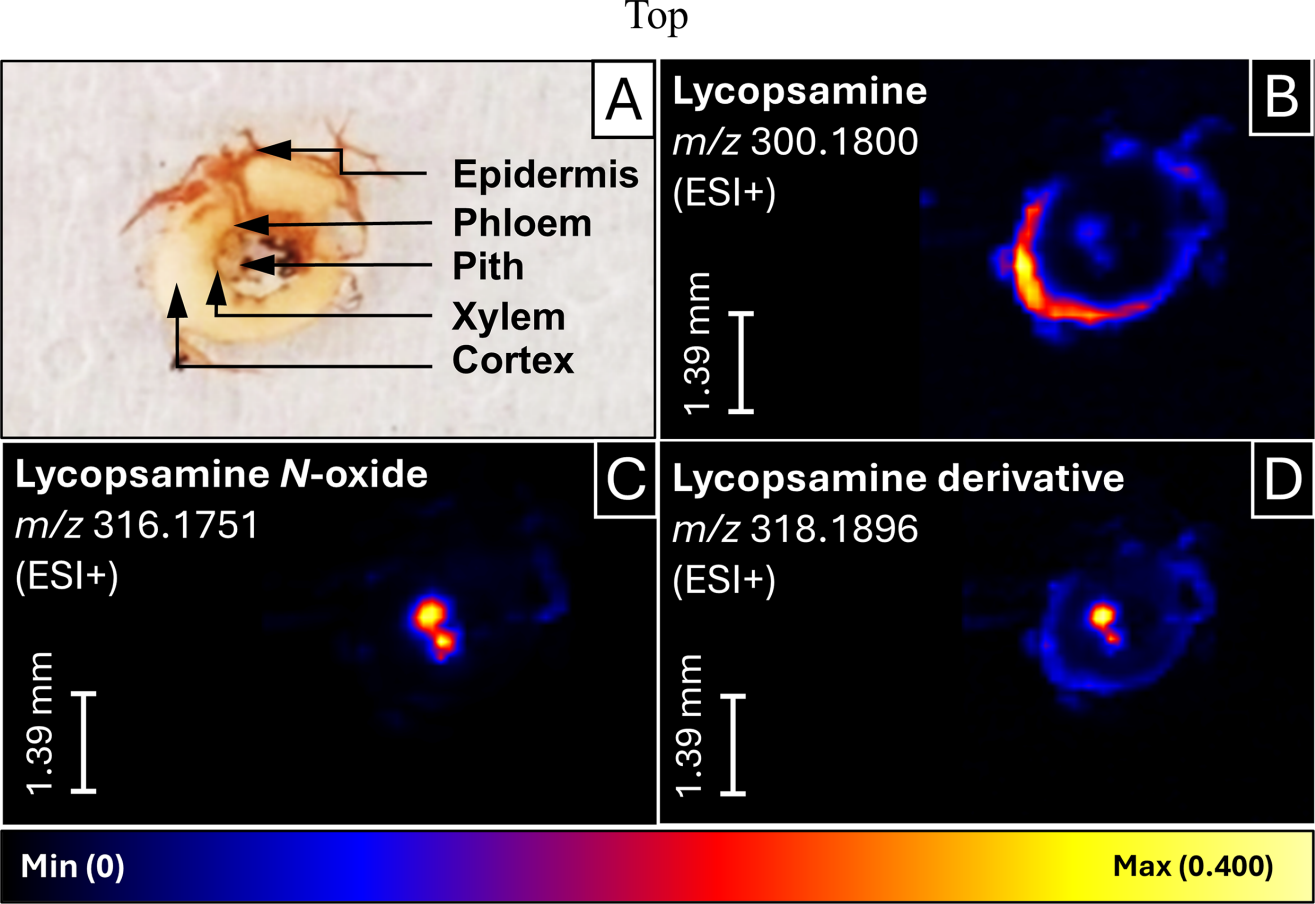
**(A)** transverse section of the stem of *L. fruticosus*, showing the different anatomical regions of interest (epidermis, cortex, xylem, phloem, and pith). DESI-MSI images of transverse stem tissues from *L. fruticosus*, showing the spatial distribution of **(B)** lycopsamine, **(C)** lycopsamine *N*-oxide and **(D)** its derivatives across anatomical regions (epidermis, cortex, xylem, phloem, and pith). Ion intensity signals were normalized to the total ion current and displayed as a heat map. Scale bar = 1.39 mm.

A transverse section of the stem of *L. fruticosus* (**Figure 5A**) was directly analyzed in positive ionization mode for the targeted analysis of PAs. Studies from Bedane et al. (2020) and Kgosana et al. (2025) have previously reported the presence of PAs in the leaf and sprig extracts of *L. fruticosus*. Lodama et al. (2016) also reported the presence of PAs in the leaves of *L. fruticosu*s. Given the risks and concerns associated with *L. fruticosus* consumption, ongoing studies and regulations of the plants used in traditional medicine are required (Mädge et al., 2020). While the differentiation of PA isomers remains challenging without MS/MS confirmation, the presence of these compounds in *L. fruticosus* is well established. Our previous work on this species confirmed and quantified multiple PAs, including lycopsamine and its derivatives, using UPLC-MS with reference standards, with concentrations ranging from 53 to 169 mg/kg across different populations (Kgosana et al., 2025).

Lycopsamine (**Figure 5B**, *m/z* 300.1800) displayed a uniform distribution with higher relative intensity around cortex of the stem. The cortex contains parenchyma cells, which are metabolically active and capable of storing alkaloids. The cortex can function as a storage unit for toxic compounds, including the pyrrolizidine alkaloids, keeping them accessible for herbivore deterrence but away from critical vascular tissues. Lycopsamine-*N*-oxide (**Figure 5C**; *m/z* 316.1751) was distributed in the xylem of the stem. Xylem is responsible for water and mineral transport from the roots to the aerial parts of the plant. The localization of lycopsamine *N*-oxide within the xylem is consistent with its hydrophilic nature and supports the hypothesis that these compounds are transported through the xylem sap following biosynthesis, to the aerial tissues where further physiological interactions may occur (Ehmke et al., 1988; Schramm et al., 2019).

Dihydrointermedine-*N-*oxide/dihydrolycopsamine-*N*-oxide (or stereoisomer) (*m/z* 318.1896) displayed a more uniform distribution with higher relative intensity in the xylem and distributed with low relative intensity in phloem, cortex, and epidermis of the stem (**Figure 5D**; **Table 4**).

**Table 4:** Pyrrolizidine alkaloid compounds identified in a transverse section of the stem of *L. fruticosus.* Compounds were identified in positive ionisation.

Key	Calculated mass [M+H] ^+^ *m/z*	Elemental formula	Compound ID	Regions of distribution in the stem
**B**	300.1800	C_15_H_25_NO_5_	Lycopsamine	Epidermis and cortex
**C**	316.1751	C_15_H_25_NO_6_	Lycopsamine- *N*-Oxide	Xylem
**D**	318.1896	C_15_H_27_NO_6_	Dihydrointermedine-*N*-oxide/dihydrolycopsamine-*N*-oxide (or stereoisomer)	Xylem

Plants employ direct defensive mechanisms by producing of toxic compounds, such as the pyrrolizidine alkaloids, that are lethal to herbivores (Dreisbach et al., 2021). These compounds have been linked to toxicological effects, including nephrotoxicity, carcinogenesis, hepatotoxicity, genotoxicity, and mutagenicity (Jayawickreme et al., 2023).

### 3.4 Aspalathin distribution in young and mature green rooibos leaves

**Figure 6:**
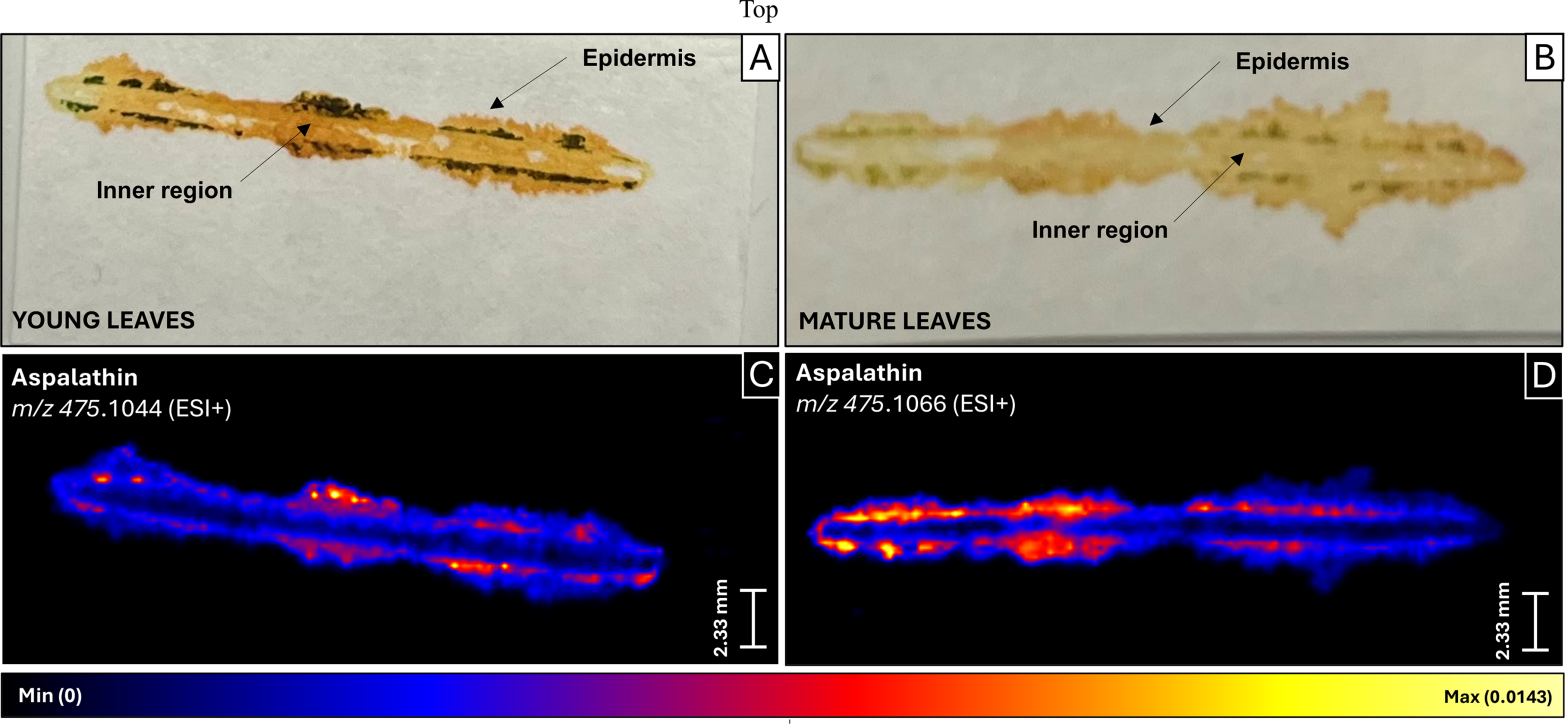
The paper imprinting and *in situ* chemical mapping of *A. linearis*. **(A)** Young leaf and **(B)** mature leaf imprints. **(C, D)** DESI–MSI ion images of the corresponding imprints showing the spatial distribution of aspalathin (*m/z* 475.1066) in positive ionisation mode as the [M+Na] ^+^ adduct. Ion intensities were normalized to the total ion current (TIC) and are displayed as heat maps. Scale bar = 2.33 mm.

Using DESI-MSI, paper imprints of *A. linearis* leaves were directly analyzed to visualize the spatial distribution of aspalathin in both unfermented young and mature green rooibos (**Figures 6A, B**). The distribution of aspalathin, a major bioactive dihydrochalcone unique to rooibos, was mapped in its sodium adduct form at *m/z* 475.1066 [M+Na] ^+^, revealing its presence across developmental stages (**Figures 6C, D**).

**Figure 7:**
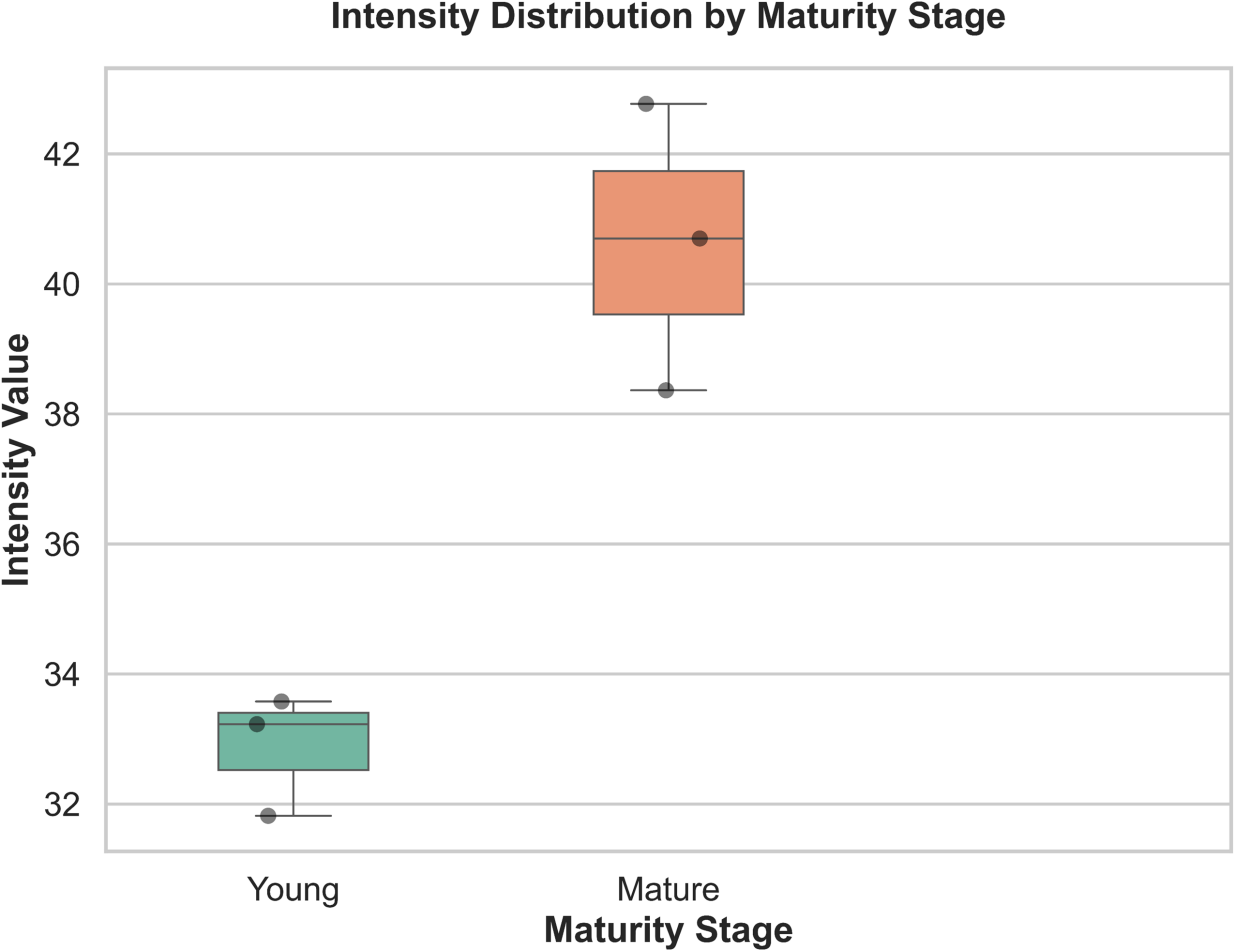
Box-and-whisker plot showing ROI-based comparison of TIC-normalised aspalathin intensity between young and mature rooibos leaves. Boxes represent the interquartile range (IQR), horizontal lines indicate the median, and whiskers represent variability across replicates.

Literature reports that *A. linearis* predominantly contains aspalathin as a chemomarker (Joubert et al., 2024). The *in situ* analysis of aspalathin on transverse and longitudinal sections of dried leaves from unfermented rooibos (*A. linearis*) was first conducted by Baranska et al. (2006) using FT-Raman spectroscopy. They reported that the aspalathin is distributed at high concentrations in the inner region of the intact leaves. Contrary to these findings, in this study, aspalathin was predominantly localized along the outer regions of the leaves. ROI-based quantification of TIC-normalized aspalathin signal intensity was performed to support the visual interpretation of ion images. The results (**Figure 7**) demonstrate a clear increase in aspalathin signal intensity in mature leaves compared to young leaves. Mature leaf ROIs exhibited higher median intensity values, whereas young leaves showed lower intensity values. The abundance of Aspalathin has been reported to vary with developmental stage and processing conditions (Joubert et al., 2024). These findings confirm a developmental variation in aspalathin abundance and distribution between young and mature tissues.

Collectively, the results from this study highlight the capability of DESI-MSI as an ambient, spatially resolved analytical technique for *in situ* metabolite mapping across diverse plant matrices. The technique consistently enabled the visualization of tissue-specific metabolite distributions and facilitated comparative analysis across samples, demonstrating its applicability for linking chemical localization with biological context. These findings highlight the versatility of DESI-MSI as a platform for spatial metabolomics in pharmacognosty studies.

## 4 Conclusion

The selected case studies represent complementary applications demonstrating the usefulness of DESI-MSI for integrating bioactivity mapping, chemotaxonomy, toxin localization, and quality-related metabolite distribution through spatially resolved, *in situ* chemical analysis of South African medicinal products. This study has demonstrated the combinatorial power that HPTLC-bioautography coupled with DESI-MSI can provide for identifying bioactive compounds in natural products extracts. Mapping the precise spatial distribution of metabolites, this technique enhances our understanding of phytochemistry, chemotaxonomy and in-cellular compound localization. Overall, this research establishes DESI-MSI as a valuable analytical tool for future natural product research and quality control, effectively bridging traditional ethnobotanical knowledge with innovative analytical technology. Future work will build upon this foundational proof-of-concept to include absolute quantification of these chemical markers, investigations into the metabolic interrelationships within tissues, and correlative microscopy to further link chemical distribution with plant histology.

## Data Availability

The original contributions presented in the study are included in the article/**Supplementary Material**. Further inquiries can be directed to the corresponding author.

## References

[B1] Abbassi-Ghadi N. Jones E. A. Gomez-Romero M. Golf O. Kumar S. Huang J. . (2015). A comparison of DESI-MS and LC-MS for the lipidomic profiling of human cancer tissue. J. Am. Soc Mass. Spectrom. 26, 255–264. doi: 10.1007/s13361-015-1278-8. PMID: 26466600

[B2] Abubakar A. R. Haque M. (2020). Preparation of medicinal plants: Basic extraction and fractionation procedures for experimental purposes. J. Pharm. Bioallied. Sci. 12, 1–10. doi: 10.4103/jpbs.jpbs_175_19. PMID: 32801594 PMC7398001

[B3] Amor Stander E. Williams W. Rautenbach F. Le Roes-Hill M. Mgwatyu Y. Marnewick J. . (2019). Visualization of aspalathin in rooibos (*Aspalathus linearis*) plant and herbal tea extracts using thin-layer chromatography. Molecules 24, 938–949. doi: 10.3390/molecules24050938. PMID: 30866512 PMC6429207

[B4] Baranska M. Schulz H. Joubert E. Manley M. (2006). In situ flavonoid analysis by FT-Raman spectroscopy: identification, distribution, and quantification of aspalathin in green rooibos (*Aspalathus linearis*). Anal. Chem. 78, 7716–7721. doi: 10.1021/ac061123q 17105163

[B5] Bedane K. G. Zühlke S. Spiteller M. (2020). Bioactive constituents of *Lobostemon fruticosus*: Anti-inflammatory properties and quantitative analysis of samples from different places in South Africa. S. Afr. J. Bot. 131, 174–180. doi: 10.1016/j.sajb.2020.02.016. PMID: 38826717

[B6] Bednarska K. Fecka I. (2022). Aspalathin and other rooibos flavonoids trapped α-Dicarbonyls and inhibited formation of advanced glycation end products *in vitro*. Int. J. Mol. Sci. 23, 14738–14766. doi: 10.3390/ijms232314738. PMID: 36499065 PMC9738946

[B7] Bemis K. D. Harry A. Eberlin L. S. Ferreira C. R. van de Ven S. M. Mallick P. . (2015). Cardinal: an R package for statistical analysis of mass spectrometry-based imaging experiments. Bioinformatics 31, 2418–2420. doi: 10.1093/bioinformatics/btv146. PMID: 25777525 PMC4495298

[B8] Cao M. Wu J. Zhu X. Jia Z. Zhou Y. Yu L. . (2024). Tissue distribution of metabolites in *Cordyceps cicadae* determined by DESI-MSI analysis. Anal. Bioanal. Chem. 416, 1883–1906. doi: 10.1007/s00216-024-05188-x. PMID: 38367042

[B9] Chen S. Zhang H. Wang X. Xu Y. Li X. Jiang Y. . (2024). Spatial distribution of differential metabolites in different parts of *Tetrastigma hemsleyanum* Diels et Gilg by ultrahigh-performance liquid chromatography/mass spectrometry and desorption electrospray ionization mass spectrometry imaging. Arab. J. Chem. 17, 105900–105911. doi: 10.1016/j.arabjc.2024.105900. PMID: 38826717

[B10] Cushnie T. T. Lamb A. J. (2005). Antimicrobial activity of flavonoids. Int. J. Antimicrob. Agents 26, 343–356. doi: 10.1016/j.ijantimicag.2005.09.002. PMID: 16323269 PMC7127073

[B11] Cushnie T. T. Lamb A. J. (2011). Recent advances in understanding the antibacterial properties of flavonoids. Int. J. Antimicrob. Agents 38, 99–107. doi: 10.1016/j.ijantimicag.2011.02.014. PMID: 21514796

[B12] De Rossi L. Rocchetti G. Lucini L. Rebecchi A. (2025). Antimicrobial potential of polyphenols: Mechanisms of action and microbial responses—A narrative review. Antioxidants 14, 200. doi: 10.3390/antiox14020200. PMID: 40002386 PMC11851925

[B13] Dreisbach D. Petschenka G. Spengler B. Bhandari D. R. (2021). 3D-surface MALDI mass spectrometry imaging for visualising plant defensive cardiac glycosides in *Asclepias curassavica*. Anal. Bioanal. Chem. 413, 2125–2134. doi: 10.1007/s00216-021-03177-y. PMID: 33544161 PMC7943518

[B14] Echeverría J. Opazo J. Mendoza L. Urzúa A. Wilkens M. (2017). Structure-activity and lipophilicity relationships of selected antibacterial natural flavones and flavanones of Chilean flora. Molecules 22, 608. doi: 10.3390/molecules22040608 28394271 PMC6154607

[B15] Efferth T. Greten H. J. (2012). Quality control for medicinal plants. Med. Aromatic. Plants 1, 2167–0412. doi: 10.4172/2167-0412.1000e131

[B16] Ehmke A. von Borstel K. Hartmann T. (1988). Alkaloid *N*-oxides as transport and vacuolar storage compounds of pyrrolizidine alkaloids in *Senecio vulgaris* L. Planta 176, 83–90. doi: 10.1007/bf00392483. PMID: 24220738

[B17] Falcão S. I. Vale N. Gomes P. Domingues M. R. Freire C. Cardoso S. M. . (2013). Phenolic profiling of Portuguese propolis by LC–MS spectrometry: Uncommon propolis rich in flavonoid glycosides. Phytochem. Anal. 24, 309–318. doi: 10.1002/pca.2412 23172843

[B18] Gao H. Li Q. (2023). Study on the spatial distribution of coumarins in *Angelica dahurica* root by MALDI‐TOF‐MSI. Phytochem. Anal. 34, 139–148. doi: 10.1002/pca.3186. PMID: 36376257

[B19] Heinrich M. Anagnostou S. (2017). From pharmacognosia to DNA-based medicinal plant authentication–pharmacognosy through the centuries. Plant. Med. 83, 1110–1116. doi: 10.1055/s-0043-108999. PMID: 28486742

[B20] Hemalatha R. G. Pradeep T. (2013). Understanding the molecular signatures in leaves and flowers by desorption electrospray ionization mass spectrometry (DESI MS) imaging. J. Agric. Food. Chem. 61, 7477–7487. doi: 10.1021/jf4011998. PMID: 23848451

[B21] Huang M. Z. Cheng S. C. Cho Y. T. Shiea J. (2011). Ambient ionization mass spectrometry: a tutorial. Anal. Chim. Acta 702, 1–15. doi: 10.1016/j.aca.2011.06.017. PMID: 21819855

[B22] Jayawickreme K. Świstak D. Ozimek E. Reszczyńska E. Rysiak A. Makuch-Kocka A. . (2023). Pyrrolizidine alkaloids—Pros and cons for pharmaceutical and medical applications. Int. J. Mol. Sci. 24, 16972–17001. doi: 10.3390/ijms242316972. PMID: 38069294 PMC10706944

[B23] Jiang Y. L. Xu Z. J. Cao Y. F. Wang F. Chu C. Zhang C. . (2021). HPLC fingerprinting-based multivariate analysis of chemical components in *Tetrastigma Hemsleyanum* Diels et Gilg: Correlation to their antioxidant and neuraminidase inhibition activities. J. Pharm. Biomed. Anal. 205, 114314–114325. doi: 10.1016/j.jpba.2021.114314. PMID: 34416550

[B24] Joubert E. D. B. D. de Beer D. (2011). Rooibos (*Aspalathus linearis*) beyond the farm gate: From herbal tea to potential phytopharmaceutical. S. Afr. J. Bot. 77, 869–886. doi: 10.1016/b978-0-12-404738-9.00014-3. PMID: 38826717

[B25] Joubert E. Human C. de Beer D. (2024). “ Natural variation in the phenolic composition of rooibos and changes during production of herbal tea and other products,” in Natural products in beverages: botany, phytochemistry, pharmacology and processing, 217–242. Cham: Springer International Publishing.

[B26] Kasote D. Ahmad A. Chen W. Combrinck S. Viljoen A. (2015). HPTLC-MS as an efficient hyphenated technique for the rapid identification of antimicrobial compounds from propolis. Phytochem. Lett. 11, 326–331. doi: 10.1016/j.phytol.2014.08.017. PMID: 38826717

[B27] Kasote D. Suleman T. Chen W. Sandasi M. Viljoen A. van Vuuren S. (2014). Chemical profiling and chemometric analysis of South African propolis. Biochem. Syst. Ecol. 55, 156–163. doi: 10.1016/j.bse.2014.03.012. PMID: 38826717

[B28] Kayser O. Kolodziej H. (1997). Antibacterial activity of extracts and constituents of *Pelargonium sidoides* and *Pelargonium reniforme*. Plant. Med. 63, 508–510. doi: 10.1055/s-2006-957752. PMID: 9434601

[B29] Kgosana M. R. Sandasi M. Ncube E. Vermaak I. Gouws C. Viljoen A. M. (2025). Exploring the wound healing potential of *Lobostemon fruticosus* using *in vitro* and *in vivo* bioassays. J. Ethnopharmacol. 336, 118632–118647. doi: 10.1016/j.jep.2024.118632. PMID: 39069028

[B30] Kharsany K. (2019). The antimicrobial properties of the major compounds found in South African propolis (Johannesburg (South Africa: University of the Witwatersrand).

[B31] Kolodziej H. (2007). Fascinating metabolic pools of *Pelargonium sidoides* and *Pelargonium reniforme*, traditional and phytomedicinal sources of the herbal medicine Umckaloabo®. Phytomedicine 14, 9–17. doi: 10.1016/j.phymed.2006.11.021. PMID: 17188477

[B32] Kumara P. M. Shaanker R. U. Pradeep T. (2019). UPLC and ESI-MS analysis of metabolites of *Rauvolfia tetraphylla* L. and their spatial localization using desorption electrospray ionization (DESI) mass spectrometric imaging. Phytochemistry 159, 20–29. doi: 10.1016/j.phytochem.2018.11.009. PMID: 30562679

[B33] Lodama K. E. Du Toit E. S. Steyn J. M. Araya H. T. Prinsloo G. Du Plooy C. P. (2016). “ Breaking seed dormancy in *Lobostemon fruticosus*”, in: VII International Symposium on Seed, Transplant and Stand Establishment of Horticultural Crops-SEST2016 1204, 115–122.

[B34] Müller T. Oradu S. Ifa D. R. Cooks R. G. Kräutler B. (2011). Direct plant tissue analysis and imprint imaging by desorption electrospray ionization mass spectrometry. Anal. Chem. 83, 5754–5761. doi: 10.1021/ac201123t 21675752 PMC3137229

[B35] Mädge I. Gehling M. Schöne C. Winterhalter P. These A. (2020). Pyrrolizidine alkaloid profiling of four *Boraginaceae* species from Northern Germany and implications for the analytical scope proposed for monitoring of maximum levels. Food. Addit. Contam.: Part. A. 37, 1339–1358. doi: 10.1080/19440049.2020.1757166 32436779

[B36] Maree J. E. Viljoen A. M. (2011). Fourier transform near-and mid-infrared spectroscopy can distinguish between the commercially important *Pelargonium sidoides* and its close taxonomically *P. reniforme*. Vib. Spectrosc. 55, 146–152. doi: 10.1016/j.vibspec.2010.10.005. PMID: 38826717

[B37] Maree J. E. Viljoen A. M. (2012). Phytochemical distinction between *Pelargonium sidoides* and *Pelargonium reniforme*—A quality control perspective. S. Afr. J. Bot. 82, 83–91. doi: 10.1016/j.sajb.2012.07.007. PMID: 38826717

[B38] Mativandlela S. P. N. Lall N. Meyer J. J. M. (2006). Antibacterial, antifungal and antitubercular activity of (the roots of) *Pelargonium reniforme* (CURT) and *Pelargonium sidoides* (DC)(Geraniaceae) root extracts. S. Afr. J. Bot. 72, 232–237. doi: 10.1016/j.sajb.2005.08.002. PMID: 38826717

[B39] Melliou E. Chinou I. (2004). Chemical analysis and antimicrobial activity of Greek propolis. Plant. Med. 70, 515–519. doi: 10.1055/s-2004-827150. PMID: 15229802

[B40] Mulaudzi N. (2020). Chemical profiling of valuable south African medicinal plants using high-performance thin layer chromatography (Pretoria, South Africa: Tshwane University of Technology).

[B41] Panara A. Aalizadeh R. Thomaidis N. S. (2022). Chemical characterisation of *Pelargonium sidoides* root based on LC‐QToF‐MS non‐target screening strategies. Phytochem. Anal. 33, 40–56. doi: 10.1002/pca.3059. PMID: 34021648

[B42] Parrot D. Papazian S. Foil D. Tasdemir D. (2018). Imaging the unimaginable: desorption electrospray ionization–imaging mass spectrometry (DESI-IMS) in natural product research. Plant. Med. 84, 584–593. doi: 10.1055/s-0044-100188. PMID: 29388184 PMC6053038

[B43] Pellati F. Orlandini G. Pinetti D. Benvenuti S. (2011). HPLC-DAD and HPLC-ESI-MS/MS methods for metabolite profiling of propolis extracts. J. Pharm. Biomed. Anal. 55, 934–948. doi: 10.1016/j.jpba.2011.03.024. PMID: 21497475

[B44] Pepeljnjak S. Kosalec I. (2004). Galangin expresses bactericidal activity against multiple-resistant bacteria: MRSA, *Enterococcus* spp. and *Pseudomonas aeruginosa*. FEMS Microbiol. Lett. 240, 111–116. doi: 10.1016/j.femsle.2004.09.018. PMID: 15500987

[B45] Przybyłek I. Karpiński T. M. (2019). Antibacterial properties of propolis. Molecules 24, 2047. doi: 10.1021/bm900088r. PMID: 31146392 PMC6600457

[B46] Qasaymeh R. M. M. (2021). Phytochemical, biological, and molecular docking studies on Fraxinus excelsior, Stachys arabica, Pelargonium sidoides, and Pelargonium reniforme (Glasgow, (United Kingdom: University of Strathclyde science).

[B47] Rahman M. M. Richardson A. Sofian-Azirun M. (2010). Antibacterial activity of propolis and honey against *Staphylococcus aureus* and *Escherichia coli*. Afr. J. Microbiol. Res. 4, 1872–1878.

[B48] Rankin-Turner S. Sears P. Heaney L. M. (2023). Applications of ambient ionization mass spectrometry in 2022: An annual review. Anal. Sci. Adv. 4, 133–153. doi: 10.1002/ansa.202300004. PMID: 38716065 PMC10989672

[B49] Rasul A. Millimouno F. M. Ali Eltayb W. Ali M. Li J. Li X. (2013). Pinocembrin: a novel natural compound with versatile pharmacological and biological activities. BioMed. Res. Int. 2013, 379850. doi: 10.1155/2013/379850. PMID: 23984355 PMC3747598

[B50] Ristivojević P. Dimkić I. Trifković J. Berić T. Vovk I. Milojković-Opsenica D. . (2016). Antimicrobial activity of Serbian propolis evaluated by means of MIC, HPTLC, bioautography and chemometrics. PloS One 11, 157097–157112. doi: 10.1371/journal.pone.0157097 PMC4896501 27272728

[B51] Sankaran S. Dubey R. Lohidasan S. (2023). Optimization of extraction conditions using response surface methodology and HPTLC fingerprinting analysis of Indian propolis. J. Biol. Act. Prod. Nat. 13, 76–93. doi: 10.1080/22311866.2023.2185681. PMID: 37339054

[B52] Schramm S. Köhler N. Rozhon W. (2019). Pyrrolizidine alkaloids: biosynthesis, biological activities, and occurrence in crop plants. Molecules 24, 498–542. doi: 10.3390/molecules24030498. PMID: 30704105 PMC6385001

[B53] Street R. A. Stirk W. A. Van Staden J. (2008). South African traditional medicinal plant trade—challenges in regulating quality, safety, and efficacy. J. Ethnopharmacol. 119, 705–710. doi: 10.1016/j.jep.2008.06.019. PMID: 18638533

[B54] Suleman T. (2015). The antimicrobial and chemical properties of South African propolis (Johannesburg (South Africa: University of the Witwatersrand).

[B55] Tillner J. Wu V. Jones E. A. Pringle S. D. Karancsi T. Dannhorn A. . (2017). Faster, more reproducible DESI-MS for biological tissue imaging. J. Am. Soc Mass. Spectrom. 28, 2090–2098. doi: 10.1007/s13361-017-1714-z. PMID: 28620847 PMC5594051

[B56] Viljoen A. M. Zhao J. Sandasi M. Chen W. Khan I. A. (2015). Phytochemical distinction between *Pelargonium sidoides* ('Umckaloabo') and *P. reniforme* through H-NMR and UHPLC-MS metabolomic profiling. Metabolomics 11, 594–602. doi: 10.1016/b978-0-323-99794-2.00015-5. PMID: 38826717

[B57] Wang J. Zhu Y. Wu C. Huang Q. (2024). Spatial distribution and comparative analysis of differential metabolites in *Curcuma longa* L. roots and rhizomes using UHPLC‐Q‐Orbitrap HRMS combined with DESI‐MSI. Phytochem. Anal. 36, 1079–1093. doi: 10.1002/pca.3493. PMID: 39723548

[B58] Widelski J. Okińczyc P. Suśniak K. Malm A. Paluch E. Sakipov A. . (2023). Phytochemical profile and antimicrobial potential of propolis samples from Kazakhstan. Molecules 28, 2984–3026. doi: 10.3390/molecules28072984. PMID: 37049747 PMC10095981

[B59] World Health Organization (2019). WHO global report on traditional and complementary medicine 2019 (Luxembourg: World Health Organization).

[B60] Wu L. Qi K. Liu C. Hu Y. Xu M. Pan Y. (2022). Enhanced coverage and sensitivity of imprint DESI mass spectrometry imaging for plant leaf metabolites by post-photoionization. Anal. Chem. 94, 15108–15116. doi: 10.1021/acs.analchem.2c03329. PMID: 36201321

[B61] Xia J. Lou G. Zhang L. Huang Y. Yang J. Guo J. . (2023). Unveiling the spatial distribution and molecular mechanisms of terpenoid biosynthesis in *Salvia miltiorrhiza* and *Salvia grandifolia* using multi-omics and DESI–MSI. Hortic. Res. 10, 109–123. doi: 10.1093/hr/uhad109. PMID: 37577405 PMC10419090

