# Supplementary material for: DESI-MSI applications for direct (spatial) biomolecular analysis of South African natural medicinal products
Source: Front Plant Sci. 2026 Jun 3;17:1802351. doi: 10.3389/fpls.2026.1802351 (PMC13272345; doi:10.3389/fpls.2026.1802351)
Supplement: Supplementary file 1 [file Table1.docx]

Supplementary Material

# Sample preparation

The plant materials were grounded, and 5 g was weighed. A volume of 50.0 mL of methanol was added and sonicated for 30 minutes. This process was repeated three times, followed by filtration through munktell filter paper. The filtrates were evaporated to dryness under vacuum at 40°C using a vacuum evaporator. The extracts of 1 mg/mL were prepared with a high-grade methanol and filtered through a 0.22 syringe filter. The filtrate was collected for UPLC-MS analysis.

# UPLC-MS method

## Propolis

The analysis was performed using a Waters® Acquity Ultra Performance Liquid Chromatographic (UPLC) system with photodiode array (PDA) detector (Waters®, Milford, MA, USA) coupled with quadrupole Time-of-Flight (Waters Xevo® G2 QToF) mass spectrometer. A volume of 1.0 μL of crude extracts (1 mg/mL) was introduced into the chromatographic system (full-loop injection) using the autosampler. The chromatographic method was completed on an Acquity® UPLC BEH C_18_ column (150 mm × 2.1 mm, i.d., 1.7 μm particle size, Waters) maintained at 50 °C. The mobile phase consisted of 0.1% formic acid in water (solvent A) and 0.1% formic acid in acetonitrile (solvent B) at a flow rate of 0.35 mL/min. The gradient elution was executed as follows: 90% A: 10% B, to 20% A: 80% B in 12 min, changed to 10% A: 90% B for 1 min, then returned to the initial ratio in 0.5 min. The system equilibrated for 1.5 min and the total run time was 15 min. Data was collected and processed using Masslynx ^TM^ v 4.2. Mass spectrometry was carried out in the negative electrospray mode. Nitrogen was used as the desolvation gas at a flow rate of 600 L/Hr, while maintaining a desolvation temperature of 400 °C. The source temperature was 100 °C. The capillary and cone voltages were set to 2500 and 40 V, respectively. Data, collected over the range *m/z* 100 to 1000, were continuum during acquisition using independent reference lock-mass ions via the LockSpray^TM^ interface to ensure mass accuracy and reproducibility. The BPI and XIC were generated.

## *Pelargonium species*

The analysis was carried out based on the method outlined in section 2.1, with modified parameters. The chromatographic conditions were: column temperature, 40 °C; total run time, 10 min. The gradient elution program was carried as follows: 95% A: 5% B, to 50% A: 50% B in 3.5 min, changed to 100% B for 3.5 min, held for 1 min, then returned to the initial ratio in 0.5 min. Mass spectrometry analysis was performed in the negative electrospray mode, the source parameters utilized were: desolvation gas flow rate, 500 L/Hr; desolvation temperature, 350°C; source temperature, 100 °C; capillary cone, 2500 V; and cone voltage, 40 V.

## *Lobostemon fruticosus*

The analysis was carried out based on the method outlined in section 2.1, with modified parameters. The chromatographic conditions were: column temperature, 50 °C; total run time, 14 min. The gradient elution program was carried as follows: 90% A: 10% B (held for 1 min), to 50% A: 50% B in 9 min, changed to 10% A: 90% B for 2 min, then returned to the initial ratio in 0.5 min. Mass spectrometry analysis was performed in the positive electrospray mode, the source parameters utilized were: desolvation gas flow rate, 600 L/Hr; desolvation temperature, 350°C; source temperature, 120 °C; capillary cone, 3000 V; and cone voltage, 30 V.

## *Aspalathus linearis*

The analysis was carried out based on the method outlined in section 2.1, with modified parameters. The chromatographic conditions were: column temperature, 40 °C; total run time, 15 min. The gradient elution program was carried as follows: 90% A: 10% B, to 20% A: 80% B in 12.5 min, held for 0.5 min, then returned to the initial ratio in 0.5 min. Mass spectrometry analysis was performed in the positive electrospray mode, the source parameters utilized were: desolvation gas flow rate, 550 L/Hr; desolvation temperature, 350°C; source temperature, 100 °C; capillary cone, 3000 V; and cone voltage, 38 V.

# The Minimum Inhibitory Concentration Assay

## Sample preparation

A weighed quantity of macerated propolis was submerged in absolute ethanol at 1:10 of sample and ethanol, respectively. The extracts were placed in a shaker incubator (Labcon) at 37°C for 7 days. The excess ethanol was evaporated, and the extract dried at room temperature. The dry ethanolic extract of propolis (EEP) will be dissolved in acetone to a starting concentration of 25 mg/ml (Suleman et al., 2015) to determine the antimicrobial activity.

## The microdilution assays

The modified microdilution assay by Eloff (1998) was used for the MIC determination of S. pneumoniae. In each well of the 96 micro-titre plates, 100 μl of sterile HTM was aseptically added. Thereafter, 100 μl of each EEP was added in duplicate in the first row of the plate, and a two-fold serial dilution was carried out. The control plate was prepared following the same procedure. Ciprofloxacin (0.01 mg/ml) as a positive control, acetone (25 mg/ml) as a negative control, and HTM (culture control) were added into the control plate in duplicate (all at a volume of 100 μl), respectively. Thereafter, 100 μl of S. pneumoniae that had been standardized to a 0.5 McFarland unit was added to each well. The microtiter plates were then hermetically sealed with a sterile adhesive film (Viljoen et al., 2003) and incubated at 37°C for 18–24 hours. Subsequently, 40 μl of a solution containing p-iodonitrotetrazolium violet (INT) at a concentration of 0.4 mg/ml will be introduced into each well. The change in colour to purple-pink was observed, which indicated microbial growth. The lowest dilution with the absence of the purple-pink colour indicated the MIC end-point value, the lowest concentration at which the pathogen was inhibited. The plates were read after 6 hours. The study was performed in triplicate and on consecutive days to ensure accuracy.

# Results

## Propolis


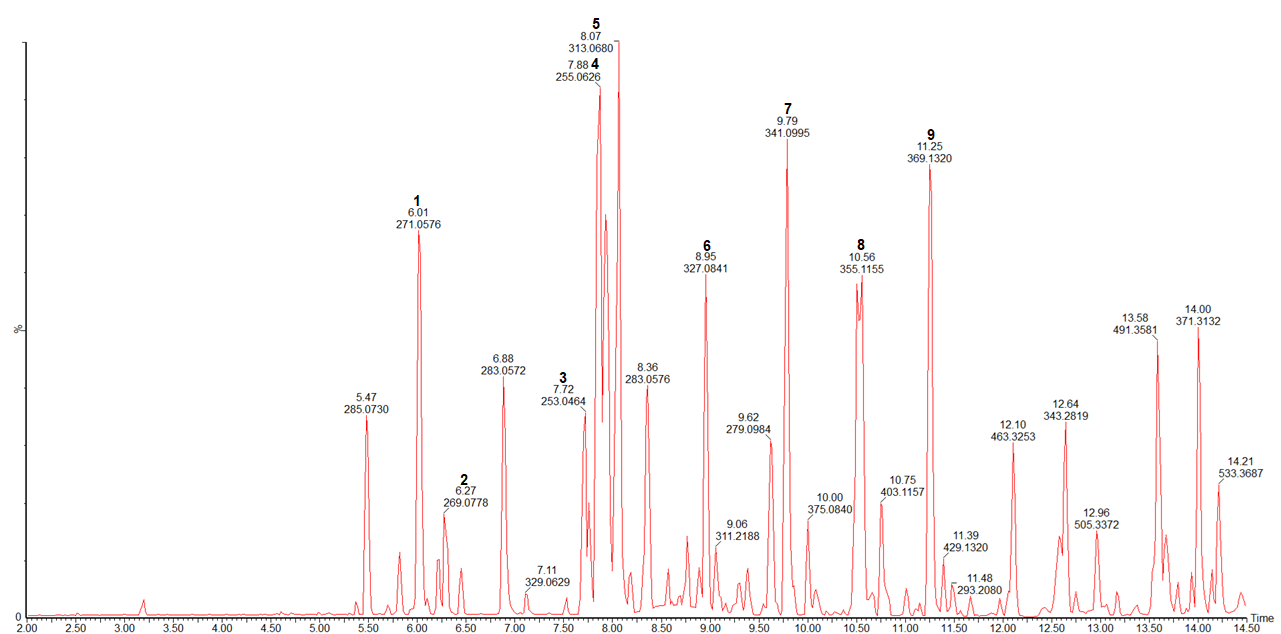


**Supplementary Figure 1:** UPLC- QToF-MS chromatogram of methanolic propolis extract.


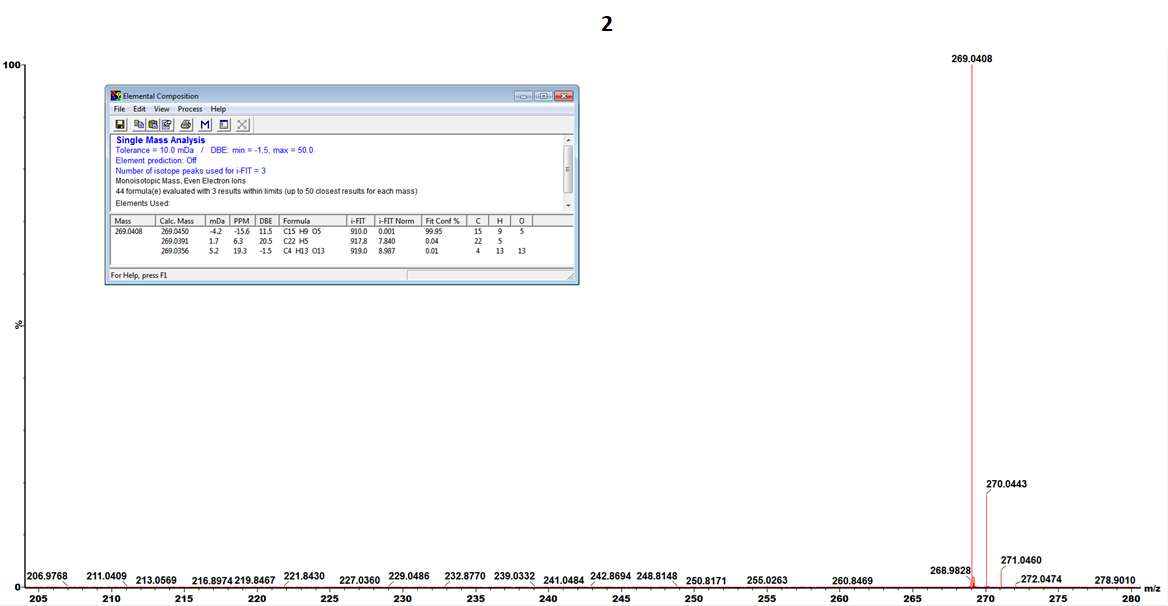


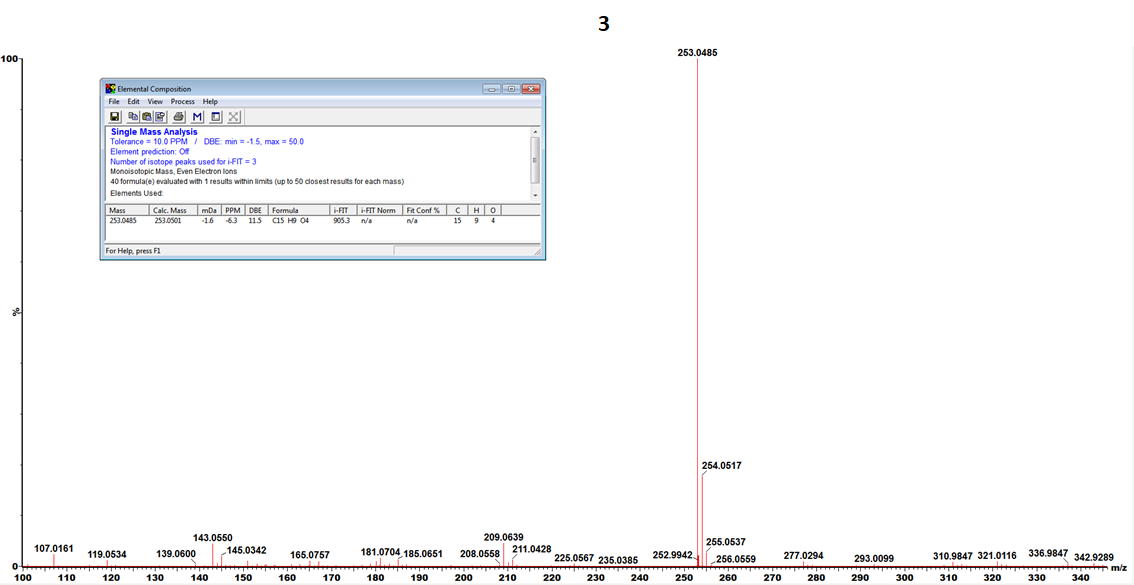


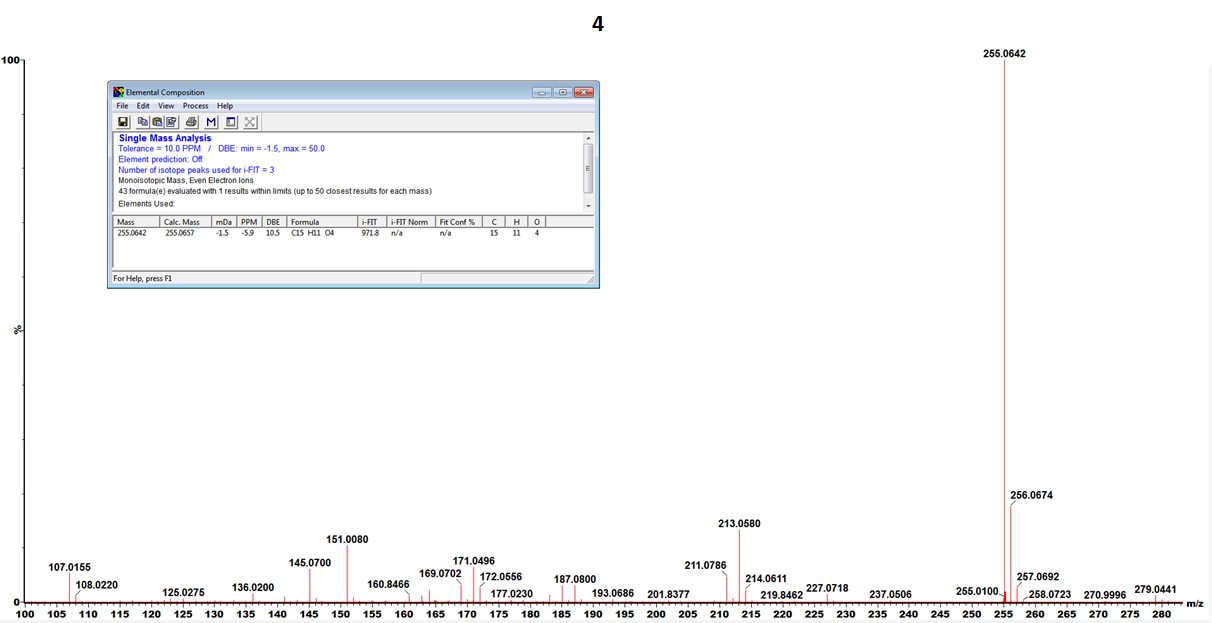


**Supplementary Figure 2:** UPLC- QToF-MS spectrum of representative bioactive compounds.

**Supplementary Table 1:** List of constituents tentatively identified in the methanolic extract of propolis using UPLC-QToF-MS.

| **Peak no**  **(DESI code)** | **Tentative identification** | **Retention time (min)** | **Molecular**  **formula** | **[M-H] ^–^ experimental (m/z)** | **[M-H] ^–^ calculated (m/z)** | **mDa** | **Fragments** |
| --- | --- | --- | --- | --- | --- | --- | --- |
| **1 (E_1_)** | Pinobanksin | 6.01 | C_15_H_12_O_5_ | 271.0576 | 271.0606 | -3.6 | 243, 165 |
| **2 (E_3_)** | Galangin | 6.27 | C_15_H_10_O_5_ | 269.0778 | 269.0450 | 33 | 255, 227 |
| **3 (E_2_)** | Chrysin | 7.72 | C_15_H_10_O_4_ | 253.0464 | 253.0501 | -3.7 | 209 |
| **4 (E_6_)** | Pinocembrin | 7.88 | C_15_H_12_O_4_ | 255.0626 | 255.0657 | -3.1 | 213, 151, 136 |
| **5 (E_4_)** | Pinobanksin acetate | 8.07 | C_17_H_14_O_6_ | 313.0680 | 313.0712 | -3.2 | 271, 253, 209, |
| **6 (E_5_)** | Pinobanksin propionate | 8.95 | C_18_H_16_O_6_ | 327.0841 | 327.0826 | -2.8 | 253, 271 |
| **7 (E_7_)** | Pinobanksin butyrate | 9.79 | C_19_H_18_O_6_ | 341.0995 | 341.1625 | -3.0 | 253, 271 |
| **8 (E_8_)** | Pinobanksin pentanoate | 10.56 | C_20_H_20_O_9_ | 355.1155 | 355.1182 | -2.8 | 253, 271 |
| **9 (E_9_)** | Pinobanksin hexanoate | 11.25 | C_21_H_22_O_6_ | 369.1320 | 369.1338 | -2.5 | 253, 271 |

**Supplementary Table 2:** The MIC value of the sample.

| **Location** | **Code** | **Average MIC value (µg/ml)** |
| --- | --- | --- |
| Muldersvlei, Stellenbosch | **WC16** | **391** |

## *Pelargonium* species


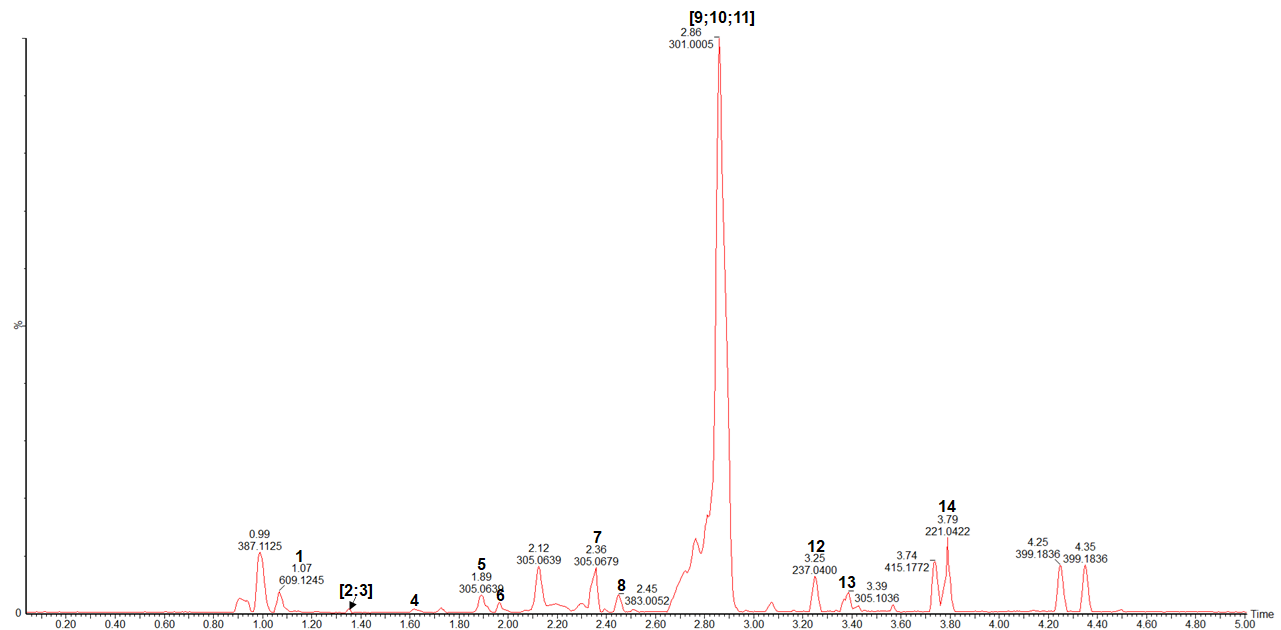


**Supplementary Figure 3:** UPLC-QToF-MS chromatogram of methanolic *Pelargonium* species extract.


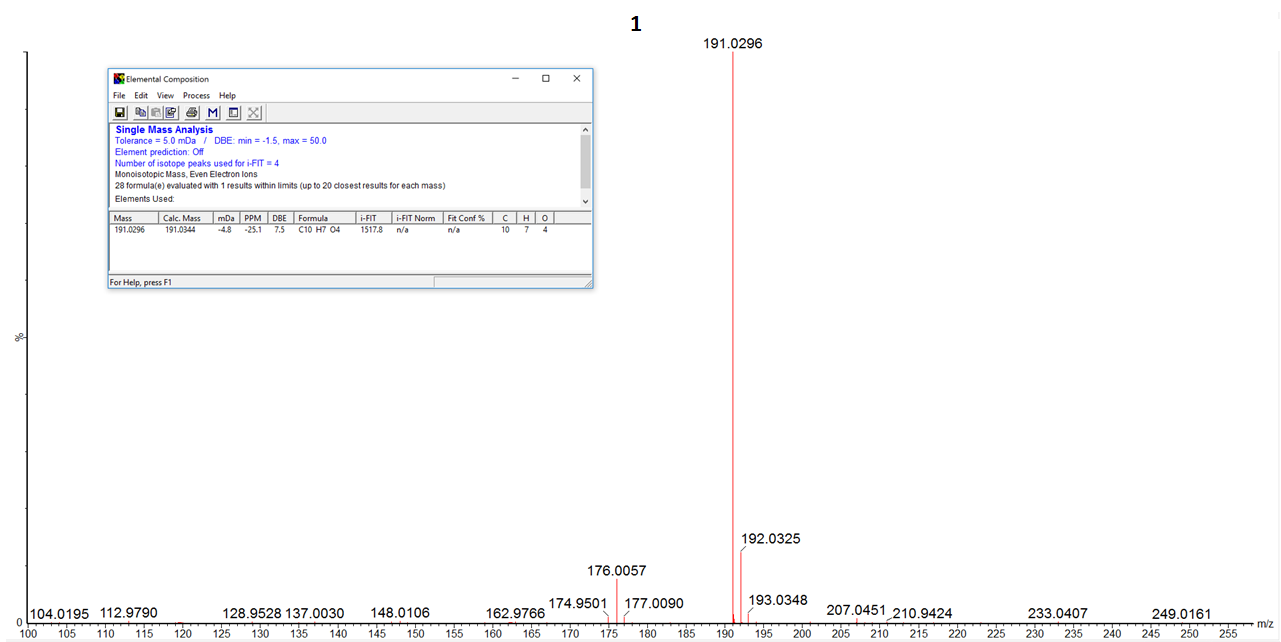


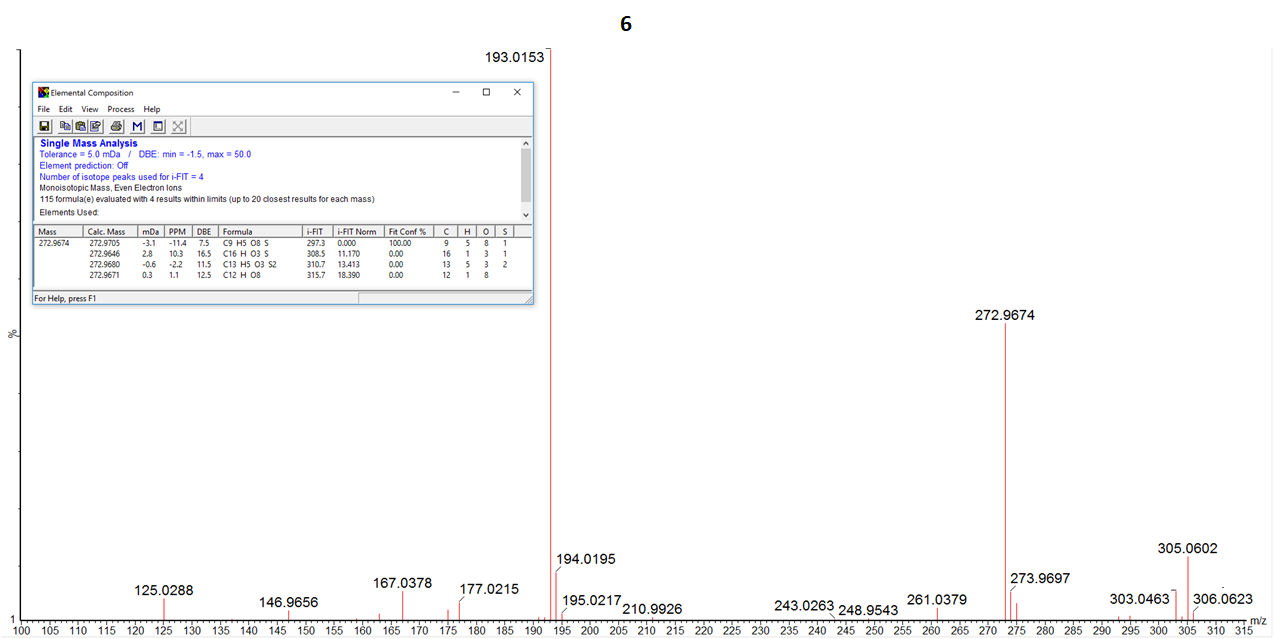


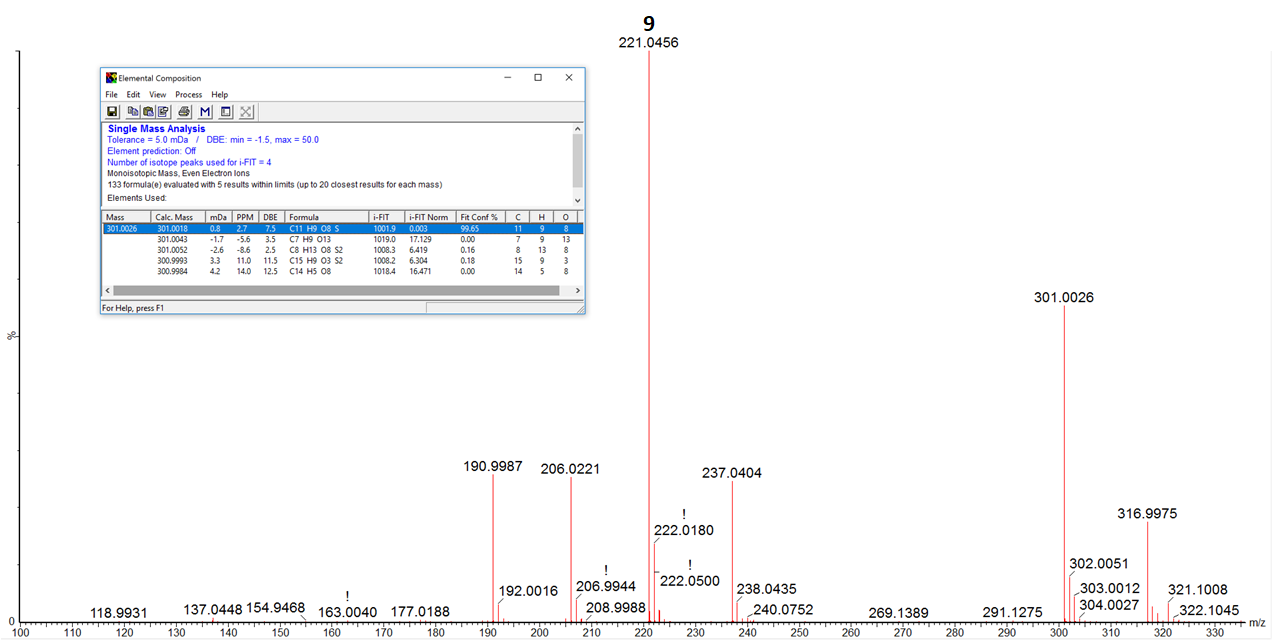


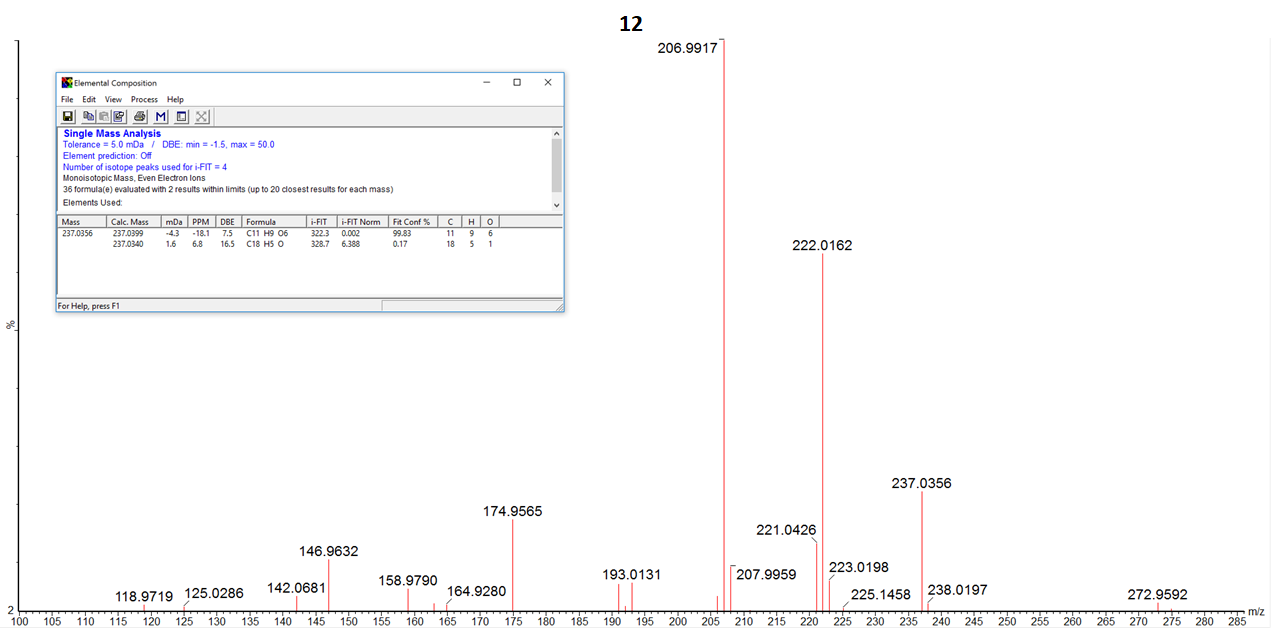


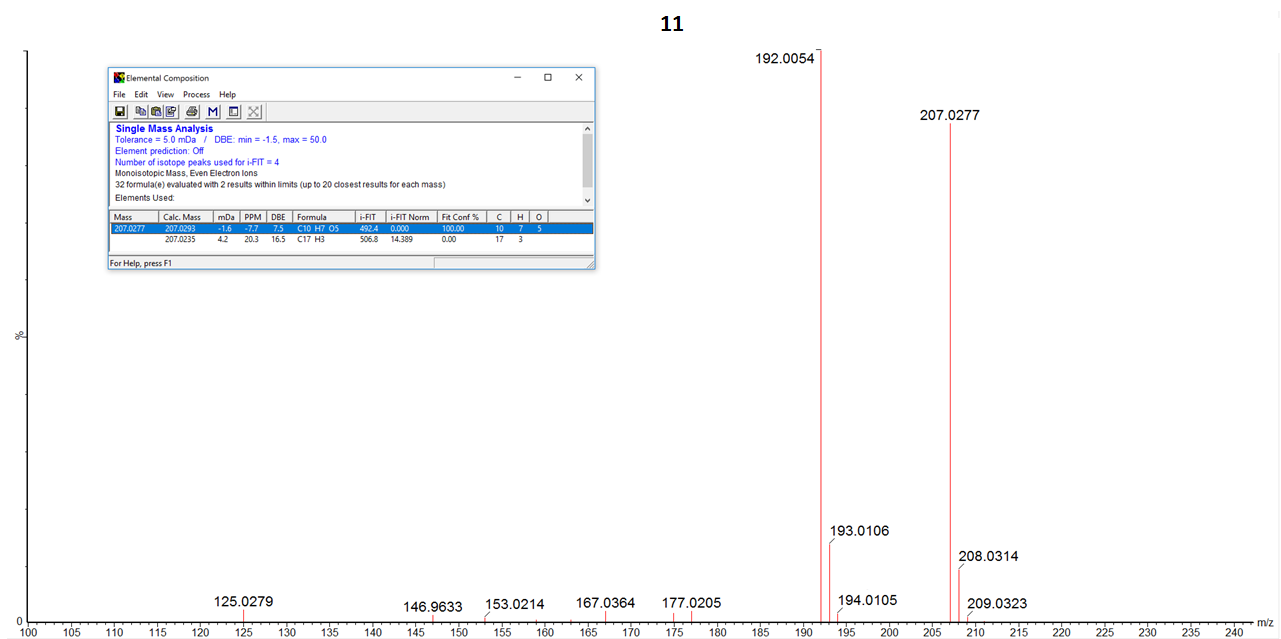


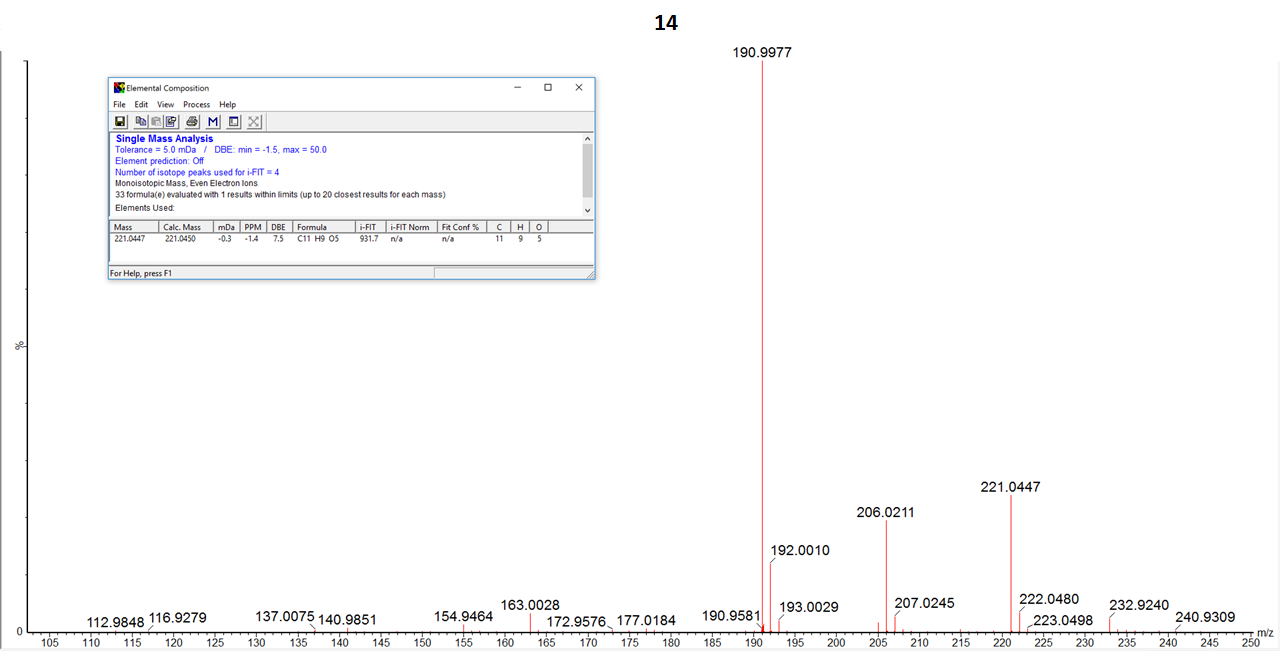


**Supplementary Figure 4:** UPLC- QToF-MS spectrum of representative chemomarkers compounds.

**Supplementary Table 3:** List of constituents tentatively identified in the methanolic extract of *Pelargonium* species using UPLC-QToF-MS.

| **Peak no**  **(DESI code)** | **Tentative identification** | **Retention time (min)** | **Molecular**  **formula** | **[M-H]^-^ exp** | **[M-H]^-^ calcd** | **mDa** | **Fragments** |
| --- | --- | --- | --- | --- | --- | --- | --- |
| **1 (A)** | Scopoletin | 1.07 | C_10_H_8_O_4_ | 191.0188 | 191.6344 | -15.6 | 148, 176 |
| **2 (K)** | cAMP | 1.36 | C_10_H_12_N_5_O_6_P | 328.0435 | 328.0447 | 19.6 | - |
| **3 (L)** | cGMP | 1.36 | C_10_H_12_N_5_O_7_P | 344.0406 | 344.0396 | 1.0 | - |
| **4 (M)** | Methyl-cGMP | 1.53 | C_11_H_14_N_5_O_7_P | 358.0054 | 358.0554 | 0.1 | - |
| **5 (G)** | Epigallocatechin | 1.89 | C_15_H_14_O_7_ | 305.0639 | 305.0678 | 1.7 | 289 |
| **6 (E)** | Dihydroxycoumarin-sulphate | 1.96 | C_9_H_6_O_8_S | 272.9702 | 272.9705 | -0.3 | - |
| **7 (I)** | Unidentified | 2.36 | - | 305.0679 | - | - | - |
| **8 (J)** | Unidentified | 2.45 | - | 383.0052 | - | - | - |
| **9 (F)** | Isofraxidin sulphate | 2.86 | C_11_H_10_O_8_S | 301.0005 | 301.0018 | -1.3 | 194, 221 |
| **10 (H)** | Hydroxy-dimethoxycoumarin-sulphate | 2.90 | C_11_H_10_O_9_S | 316.9960 | 316.9967 | -0.7 | 221 |
| **11 (B)** | Isofraxetin | 2.92 | C_10_H_8_O_5_ | 207.9848 | 207.0293 | -44 | 191 |
| **12 (D)** | Dihydroxy-dimethoxycoumarin | 3.25 | C_11_H_10_O_6_ | 237.0400 | 237.0399 | 0.1 | 207 |
| **13 (N)** | Unidentified | 3.39 | - | 305.1036 | - | - | - |
| **14 (C)** | Umckalin/Isofraxidin | 3.79 | C_11_H_10_O_5_ | 221.0422 | 221.0400 | -2.8 | 191, 206 |

## *Lobostemon fruticosus*


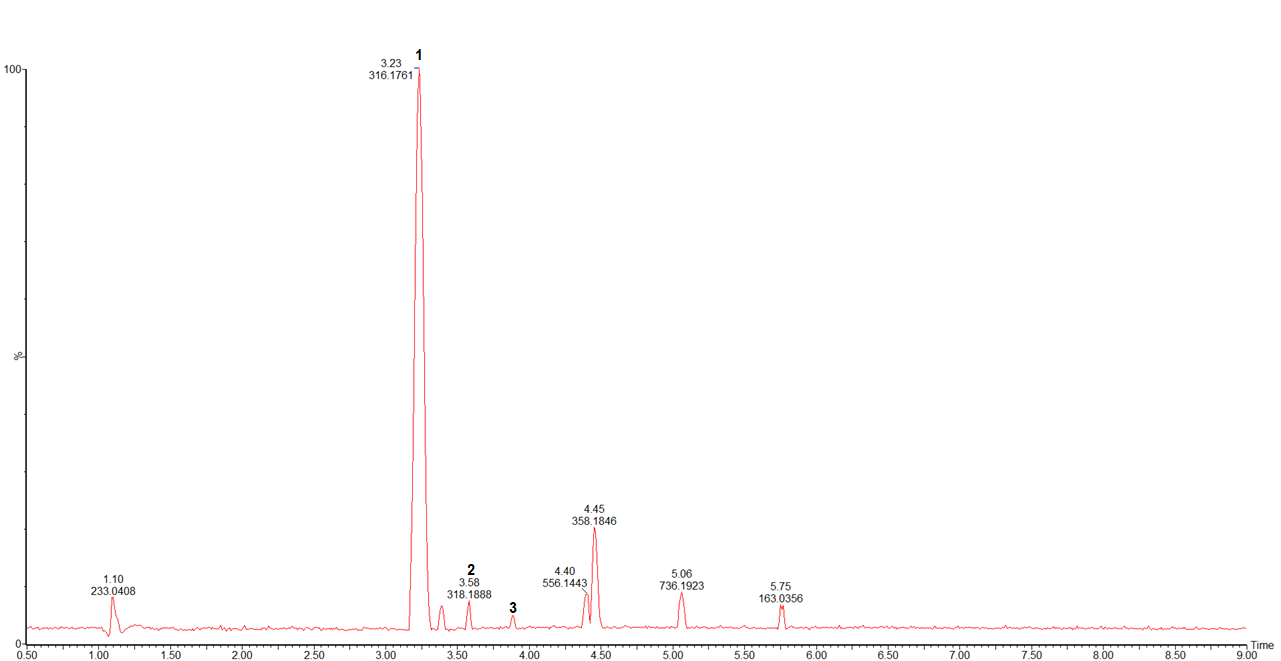


**Supplementary Figure 5:** UPLC-QToF-MS chromatogram of methanolic *Lobostemon fruticosus*.


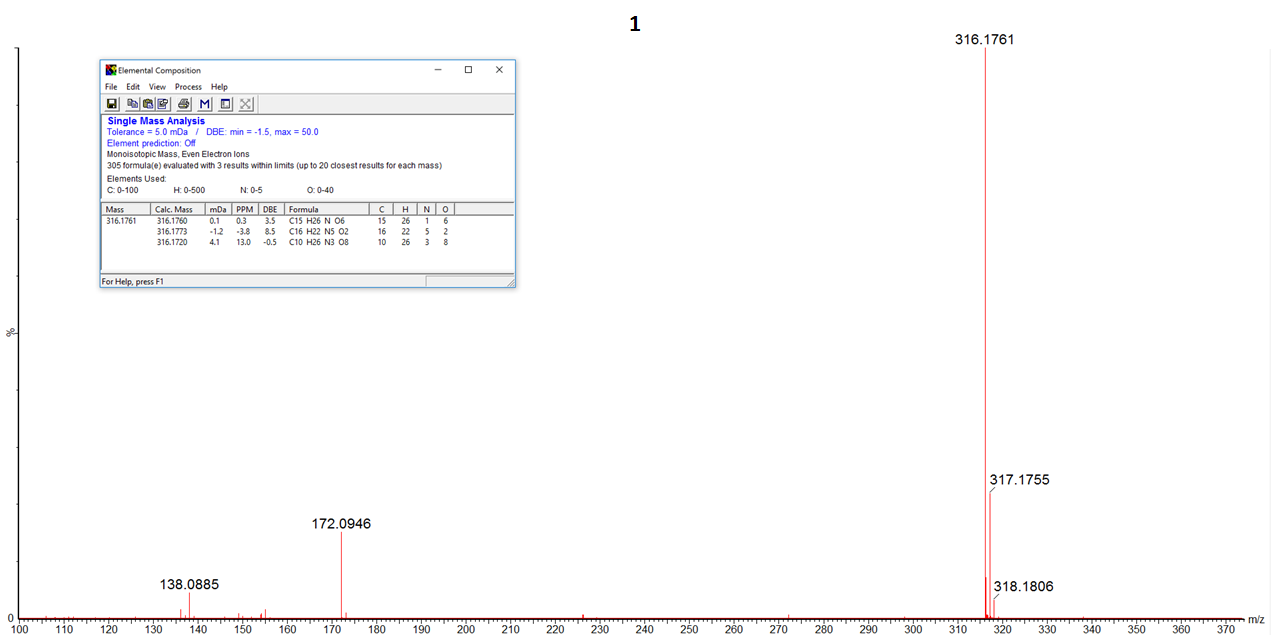


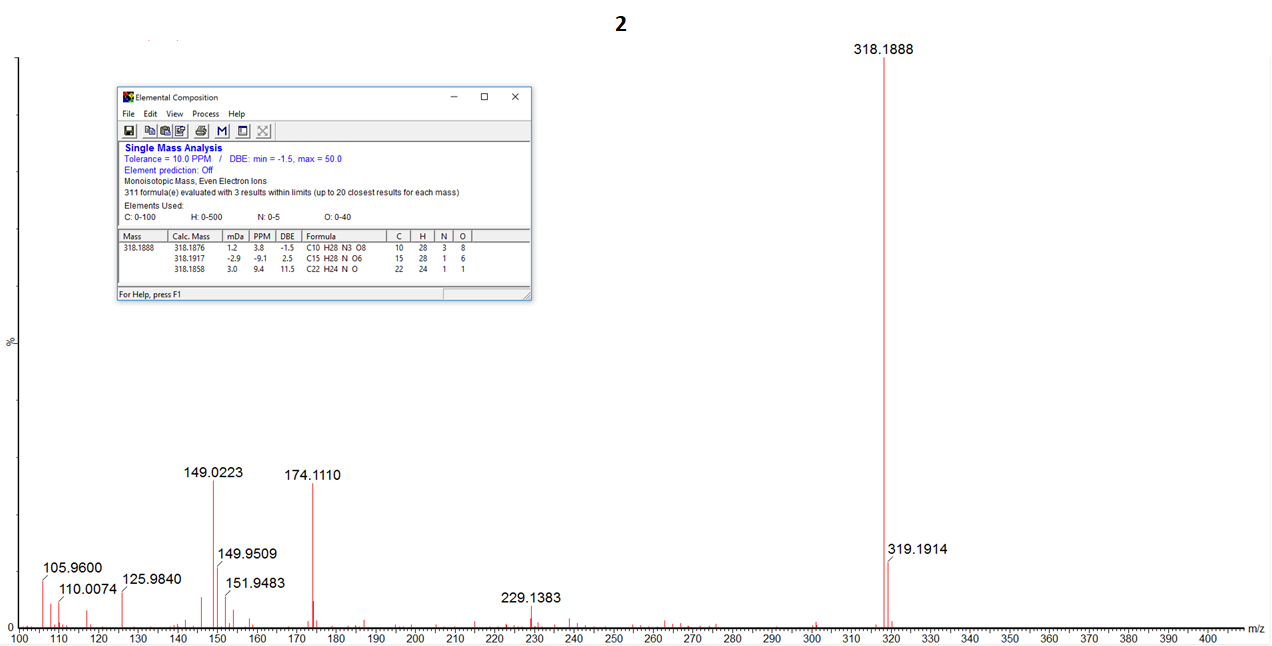


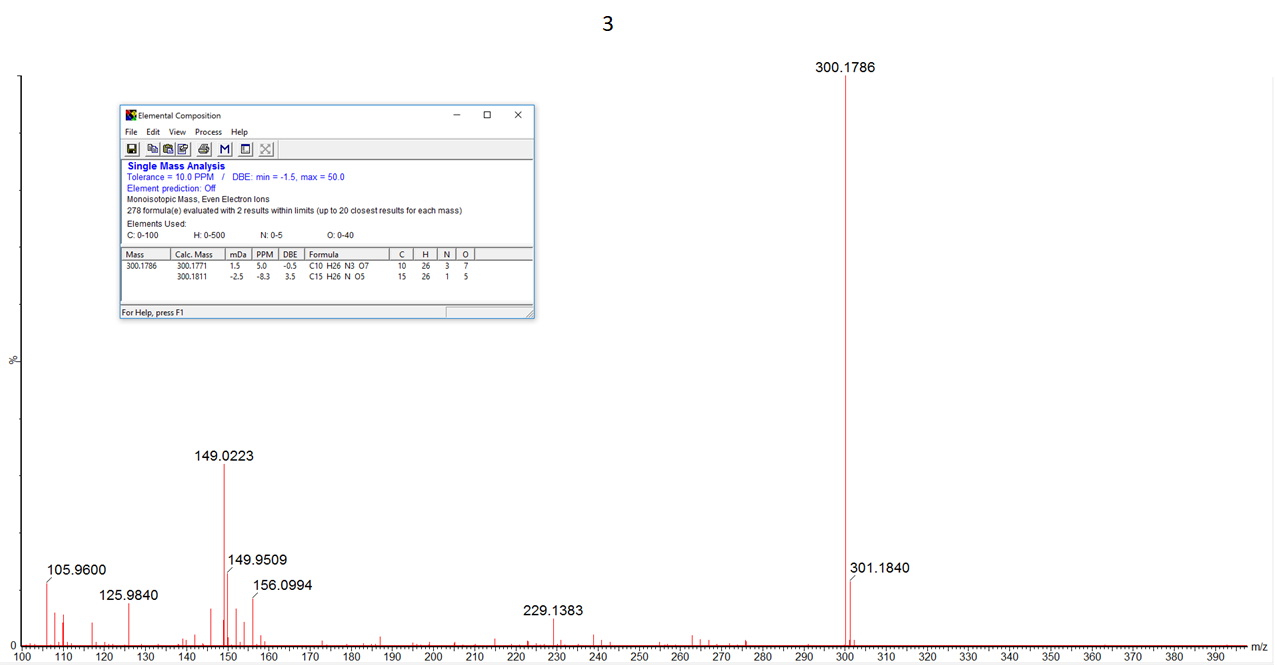


**Supplementary Figure 6:** UPLC-QToF-MS spectrum of pyrrolizidine alkaloids. **(1)** Lycopsamine- *N*-Oxide **(2)** Dihydrointermedine-*N*-oxide/dihydrolycopsamine-*N*-oxide (or stereoisomer) and **(3)** Lycopsamine.

**Supplementary Table 4:** List of constituents tentatively identified in the methanolic extract of *Lobostemon fruticosus* using UPLC-QToF-MS.

| **Peak no**  **(DESI code)** | **Tentative identification** | **Retention time (min)** | **Molecular**  **formula** | **[M+H] ^+^ experimental (m/z)** | **[M+H] ^+^ calculated (m/z)** | **mDa** | **Fragments** |
| --- | --- | --- | --- | --- | --- | --- | --- |
| **1 C** | Lycopsamine- *N*-Oxide | 3.23 | C_15_H_25_NO_6_ | 316.1761 | 316.1760 | 0.1 | 172, 138 |
| **2 D** | Dihydrointermedine-*N*-oxide/dihydrolycopsamine-*N*-oxide (or stereoisomer) | 3.58 | C_15_H_27_NO_6_ | 318.1888 | 318.1917 | -2.9 | 174, 106 |
| **3 B** | Lycopsamine | 3.88 | C_15_H_25_NO5 | 300.1786 | 300.1811 | -2.5 | 156 |

## *Aspalathus linearis*


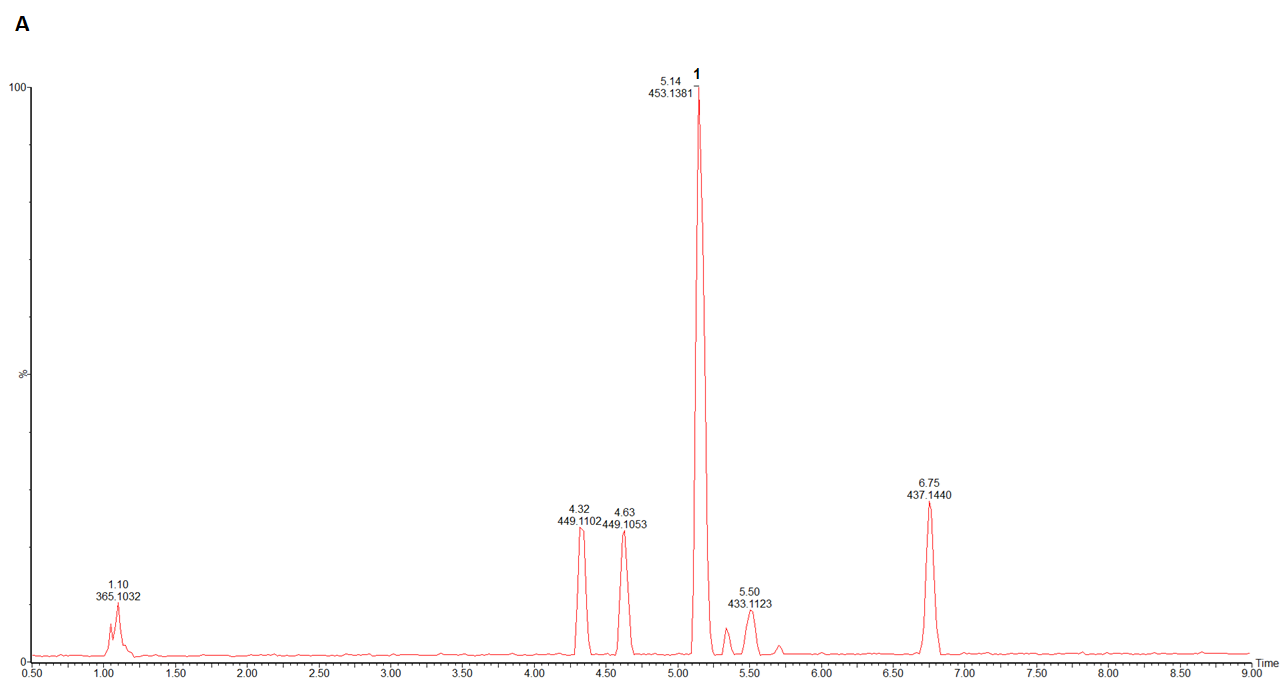


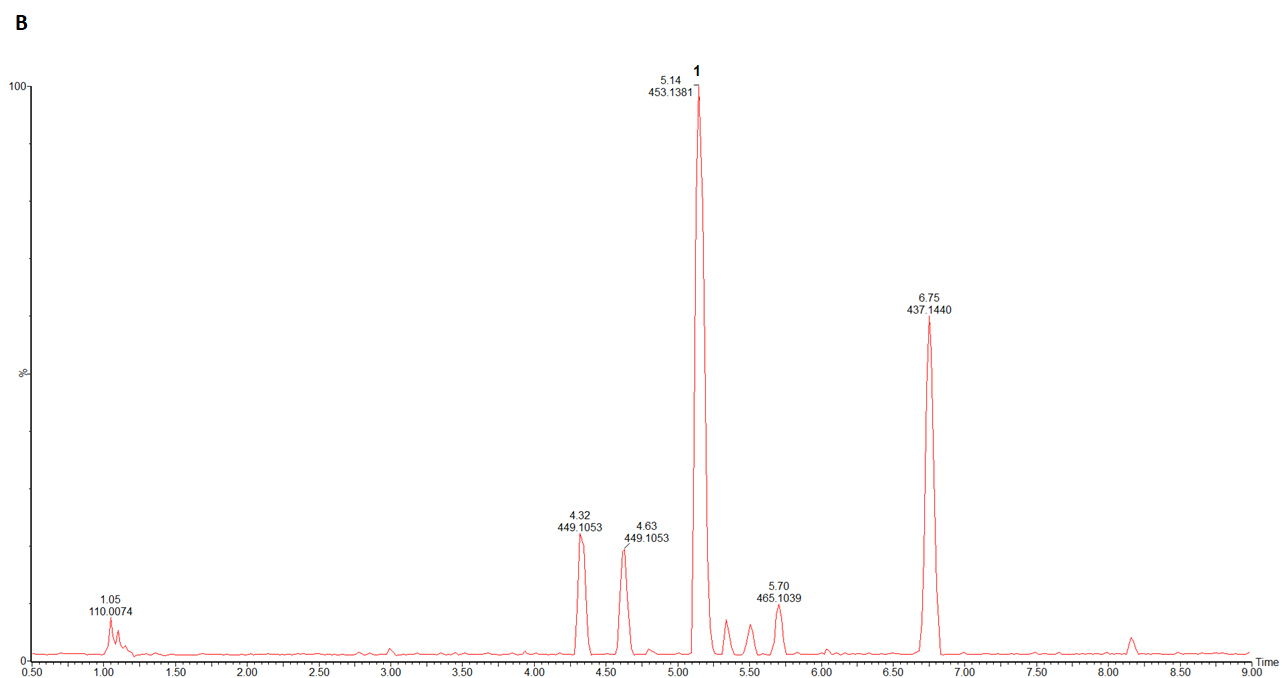


**Supplementary Figure 7:** UPLC-QToF-MS chromatogram of methanolic *Aspalathus linearis* extract. (**A**) Young and (**B**) Mature.


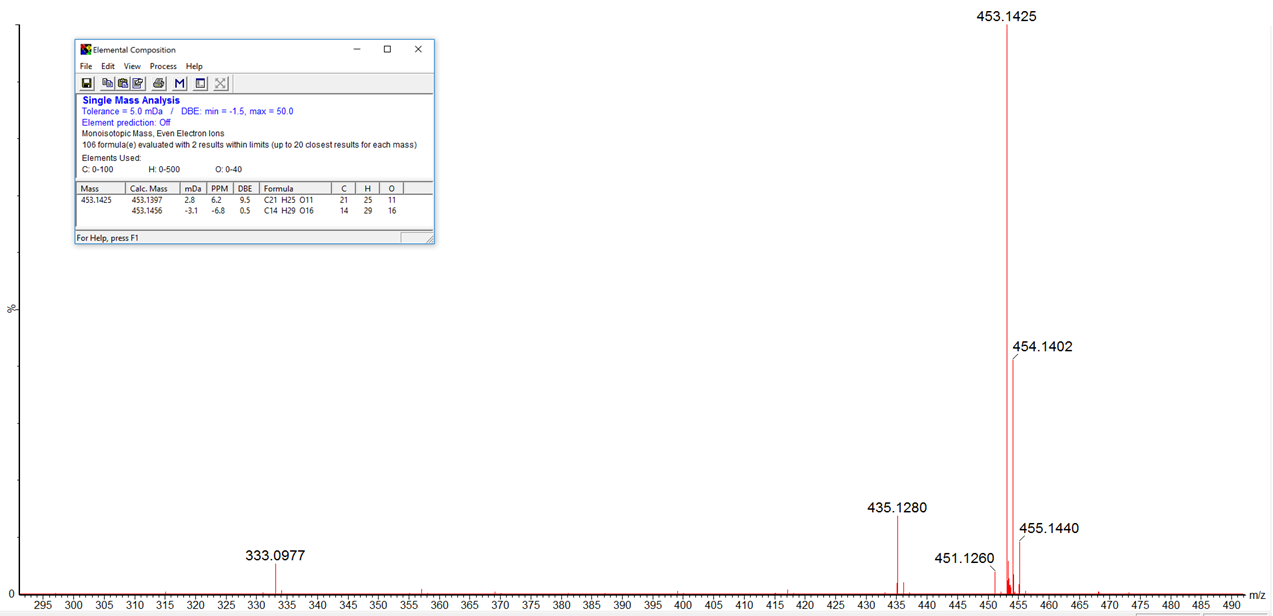


**Supplementary Figure 8:** UPLC-QToF-MS spectrum of aspalathin. (**A**) Young and (**B**) Mature.

**Supplementary Table 5:** List of constituents tentatively identified in the methanolic extract of *Aspalathus linearis* using UPLC-QToF-MS.

| **Sample** | **Tentative identification** | **Retention time (min)** | **Molecular**  **formula** | **[M+H] ^+^ experimental (m/z)** | **[M+H] ^+^ calculated (m/z)** | **mDa** | **Fragments** |
| --- | --- | --- | --- | --- | --- | --- | --- |
| **Young** | Aspalathin | 5.14 | C_21_H_24_O_11_ | 453.1381 | 453.1397 | -1.6 | 333, 303 |
| **Mature** | Aspalathin | 5.14 | C_21_H_24_O_11_ | 453.1381 | 453.1397 | -1.6 | 333, 303 |

# Reference

Eloff, J.N., 1998. A sensitive and quick microplate method to determine the minimal inhibitory concentration of plant extracts for bacteria. *Planta medica*, *64*(08), 711-713.

Suleman, T., Van Vuuren, S., Sandasi, M. and Viljoen, A.M., 2015. Antimicrobial activity and chemometric modelling of South African propolis. *Journal of applied microbiology*, *119*(4), 981-990.

Viljoen, A., Van Vuuren, S., Ernst, E., Klepser, M., Demirci, B., Başer, H. and Van Wyk, B.E., 2003. *Osmitopsis asteriscoides* (Asteraceae)-the antimicrobial activity and essential oil composition of a Cape-Dutch remedy. *Journal of Ethnopharmacology*, *88*(2-3), 137-143.
